# Role of preoperative intravenous iron therapy to correct anemia before major surgery: a systematic review and meta-analysis

**DOI:** 10.1186/s13643-021-01579-8

**Published:** 2021-01-23

**Authors:** Abdelsalam M. Elhenawy, Steven R. Meyer, Sean M. Bagshaw, Roderick G. MacArthur, Linda J. Carroll

**Affiliations:** 1 Division of Cardiac Surgery, Department of Surgery, Faculty of Medicine and Dentistry, University of Alberta, Edmonton, Alberta Canada; 2 Division of Cardiothoracic Surgery, Al Azhar University, Cairo, Egypt; 3 Division of Critical Care Medicine, Faculty of Medicine and Dentistry, University of Alberta, Edmonton, Alberta Canada; 4 School of Public Health, University of Alberta, Edmonton, Alberta Canada

**Keywords:** Intravenous iron therapy, Preoperative anemia, Major surgery

## Abstract

**Background:**

Preoperative anemia is a common comorbidity that often necessitates allogeneic blood transfusion (ABT). As there is a risk associated with blood transfusions, preoperative intravenous iron (IV) has been proposed to increase the hemoglobin to reduce perioperative transfusion; however, randomized controlled trials (RCT) investigating this efficacy for IV iron are small, limited, and inconclusive. Consequently, a meta-analysis that pools these studies may provide new and clinically useful information.

**Methods/design:**

Databases of MEDLINE, EMBASE, EBM Reviews; Cochrane-controlled trial registry; Scopus; registries of health technology assessment and clinical trials; Web of Science; ProQuest Dissertations and Theses; Clinicaltrials.gov; and Conference Proceedings Citation Index-Science (CPCI-S) were searched. Also, we screened all the retrieved reference lists.

**Selection criteria:**

Titles and abstracts were screened for relevance (i.e., relevant, irrelevant, or potentially relevant). Then, we screened full texts of those citations identified as potentially applicable.

**Results:**

Our search found 3195 citations and ten RCTs (1039 participants) that met our inclusion criteria. Preoperative IV iron supplementation significantly decreases ABT by 16% (risk ratio (RR): 0.84, 95% confidence interval [CI]: 0.71, 0.99, *p* = 0.04). In addition, preoperatively, hemoglobin levels increased after receiving IV iron (mean difference [MD] between the study groups: 7.15 g/L, 95% CI: 2.26, 12.04 g/L, *p* = 0.004) and at follow-up >  4 weeks postoperatively (MD: 6.46 g/L, 95% CI: 3.10, 9.81, *p* = 0.0002). Iron injection was not associated with increased incidence of non-serious or serious adverse effects across groups (RR: 1.13, 95% CI: 0.78, 1.65, *p* = 0.52) and (RR: 0.96, 95% CI: 0.44, 2.10, *p* = 0.92) respectively.

**Conclusions:**

With moderate certainty, due to the high risk of bias in some studies in one or two domains, we found intravenous iron supplementation is associated with a significant decrease in the blood transfusions rate, and modest hemoglobin concentrations rise when injected pre-surgery compared with placebo or oral iron supplementation. However, further full-scale randomized controlled trials with robust methodology are required. In particular, the safety, quality of life, and cost-effectiveness of different intravenous iron preparations require further evaluation.

**Supplementary Information:**

The online version contains supplementary material available at 10.1186/s13643-021-01579-8.

## Background

Preoperative anemia remains the most common hematological deficit affecting the patients undergoing major surgery. It is an independent risk factor for perioperative allogeneic blood transfusion (ABT), as revealed in a meta-analysis of 949,445 patients [1] and associated with increased postoperative morbidity and mortality [2–5]. However, the evidence to support the treatment of anemia with transfusion in a perioperative setting is still lacking and requires further research. Recently, two large transfusion trigger meta-analyses reported different results. The first study reported similar mortality rates when a restrictive or liberal transfusion strategy was applied [6]. Unfortunately, this study investigated only the mortality outcome without reporting data on the morbidity occurrence post-transfusion, and their patients’ populations are heterogeneous being a mixture of medical and surgical settings [6]. The second meta-analysis showed that the restrictive transfusion strategy was associated with less mortality and morbidity in the critical care arm of the study [7]. Interestingly, in the perioperative population, the restrictive transfusion strategy with transfusion triggers of 7–7.5 g/dL may increase the risk of mortality. However, the investigators considered this finding of less robust evidence due to being insufficiently accumulated sample size compared with the critical care population recommending further research [7].

Furthermore, several literatures reported that blood transfusion is associated with adverse outcomes perioperatively [8–10]. Furthermore, data obtained from two large multicenter databases of 23,348 patients [11], and 227,425 patients [12] demonstrated that not only severe but also moderate/mild preoperative anemia is an independent risk factor for postoperative morbidity and 30-day mortality. These findings would suggest that approaches to reduce avoidable ABT might be associated with improved postoperative outcomes.

Although it is correctable [4], iron-deficiency anemia (IDA) remains the most frequent category of anemia that develops in patients undergoing major surgery [13, 14] due to inadequate iron intake and substantial blood loss perioperatively [15]. Oral iron supplementation requires a long time to replenish the exhausted iron stores and is associated with intolerability in about 70% of patients [16]. In contrast, intravenous (IV) iron supplementation allows for a large quantity of iron to be administered over a few doses and has excellent availability for erythropoiesis [17], to increase the hemoglobin (Hb) concentration significantly in nonsurgical recent studies; a review [18], a randomized controlled trial (RCT) [19], and several meta-analyses [20–22].

Intravenous iron therapy for preoperative anemia has been tested in some RCTs. However, to date, the evidence to support its safety and efficacy is unclear. Due to the paucity of trials, most of which are of small sample size and have negative outcomes, which may be a result of being underpowered, there is insufficient evidence to support the use of IV iron to decrease ABT. A meta-analytic approach that pools these studies would aid in addressing the limitations of trial size, which would increase the power to observe statistically significant differences and may provide new and clinically useful evidence. This meta-analysis will investigate (a) the ability of parenteral iron administration to reduce ABT requirement by improving preoperative Hb concentrations, and (b) the safety of parenteral iron in terms of mortality, morbidity, and adverse events (AEs) compared with placebo/oral iron as the standard of care.

## Patients and methods

### Protocol and registration

Our study protocol has been published [23] and registered with the International Prospective Register of Systematic Reviews (PROSPERO), systematic review registration number: PROSPERO CRD42015016771) on February 16, 2015. Initially, following our published inclusion/exclusion criteria [23], we found only two eligible trials of small sample size. Consequently, to recruit more trials, we had to modify our inclusion/exclusion criteria (Additional File 1 A) and made an update on the PROSPERO website on April 27, 2017. The current study was conducted following the Preferred Reporting Items for Systematic Reviews and Meta-analyses (PRISMA) statement [24] (Additional file 1 B).

#### Eligibility criteria

To be eligible, a study had to be a randomized or quasi-randomized controlled clinical trial investigating adult surgical participants in which the intervention drug was IV iron as monotherapy, initiated/completed preoperatively. Being comprehensive, we did not apply any publication date/status or language restrictions.

#### Search strategy

With the help of a health sciences librarian, using our search strategy (Additional File 1 C), the following databases were searched: MEDLINE, EMBASE; EBM Reviews; the Cochrane-controlled trial registry in the Cochrane Library; Scopus; registries of health technology assessment and clinical trials; and Web of Science. In addition, we searched the ProQuest Dissertations and Theses database; the ClinicalTrials.gov website (National Institute of Health) for completed but unpublished studies; and Conference Proceedings Citation Index (CPCI)-Science since 1990.

The search was started from the earliest retrievable date of each database to February 2019, supplemented by a manual search of reference lists of all retrieved trials, previous reviews of related areas, and Google engine. We ran an updated search on 26 October 2020, which came up with a pilot study in cardiac surgery [25], and a trial in abdominal surgery [26], and we decided to include both in our update.

### Study screening and data extraction

Authors AE and SM conducted all study screening. Then, they reviewed the titles, abstracts, and reference lists of all included publications to retrieve potentially relevant studies. Full text of retrieved studies was subjected to a second phase of screening for eligibility as defined by the modified inclusion/exclusion criteria and study design for methodological quality. In cases of study eligibility disagreement occurred, a consensus was accomplished through discussion. The same two authors (AE and SM) reviewed the relevant studies and extracted the relevant data using a structured form as per our protocol [23].

In our published protocols, the secondary outcomes were the number of units of blood or blood products transfused perioperatively, all-cause mortality, transfusion-related acute lung injury, neurologic complications, adverse events, postoperative infections, cardiopulmonary complications, intensive care unit (ICU) admission/readmission, length of hospital stay, acute kidney injury, development of antibodies against platelets or white blood cells, post-transfusion purpura, graft vs. host disease, infection, immunomodulation, and iron overload. However, due to the absence/insufficient data from the primary trials, we were not able to achieve meta-analyses for some of the secondary outcomes.

On the other hand, other secondary outcomes that were not planned in our protocol as the health-related quality of life (HRQoL) measure as indicators for the quality of recovery post-surgery, the IDA parameters including reticulocyte (%) percentage, mean corpuscular volume (MCV), mean corpuscular hemoglobin (MCH), and mean corpuscular hemoglobin concentration (MCHC) were reported in this meta-analysis since we found such data published in the included trials.

### Assessment of the risk of bias

The Cochrane Collaboration’s tool (Additional File 1 D) was used [27] to evaluate the studies sources of bias.

### Data synthesis and analysis

For primary outcomes, proportions of study participants who received an ABT was analyzed as risk ratios (RR) with their 95% confidence intervals (CIs); in contrast, the hematopoietic response was analyzed as the mean difference (MD) with their 95% CI for the Hb concentration change with its statistical significance between groups. Safety outcomes, including mortality, infection, and treatment-related AEs, were analyzed as RR with their 95% CIs.

Where standard deviation (SD) was not reported, the trial’s authors were contacted in an effort to get the primary data to compute it. In the absence of SD information, SDs were estimated from 95% CIs, z-statistics, and *p* values or imputed using the largest reported SD from other trials [28].

The Cochran's *Q* test was used to calculate the statistical heterogeneity among studies. An *I*^2^ value ≥ 40% suggests substantial statistical heterogeneity, and a random-effects model (DerSimonian and Laird technique) [29] was appropriate. With absent or low heterogeneity (*I*^2^ value < 40%) [30], the fixed-effects model (Mantel-Haenszel technique) was appropriate, with a random-effects model as a sensitivity analysis. The *Z* test was used to test the overall effect. We conducted a sensitivity analysis on our primary efficacy outcomes and safety outcome by excluding trials with a high risk of bias for one or more key domains [30]. In the analyses, to avoid moving the pooled estimate of IV iron treatment effect closer to null, initially, all trials reporting zero-event data for both trial arms were excluded from the analysis. Then, the analysis was repeated, including these trials to identify any change in the effect estimate as a sensitivity analysis, to provide a more valid estimate by having analytic consistency, and to offer more generalizability in the clinical practice by including all the available data [31]. Across the meta-analysis, the statistical significance was set as a *p* value < 0.05.

#### Grading the strength of the evidence

We judged the overall quality of evidence for each outcome in the involved trials using the Grading of Recommendations Assessment, Development, and Evaluation (GRADE) approach and created “Summary of findings” tables using GRADE Profiler (GRADEpro GDT) [32], following the guidance in the Cochrane Handbook for Systematic Reviews of Interventions [33]. Our GRADE judgment of certainty was achieved through consideration of these five domains: risk of bias, inconsistency, indirectness, imprecision, and publication bias.

#### Subgroup analysis and exploration of heterogeneity

To assess the heterogeneity, subgroup analyses were planned when feasible by having at least two studies in at least two subgroups for the primary outcome. The approach of “leave-one-out” sensitivity analysis was implemented to assess the stability of the meta-analysis outcomes [34].

RevMan 5.3 software: The Nordic Cochrane Centre, The Cochrane Collaboration; 2014 [35] was used to perform the analysis.

## Results

**Fig. 1:**
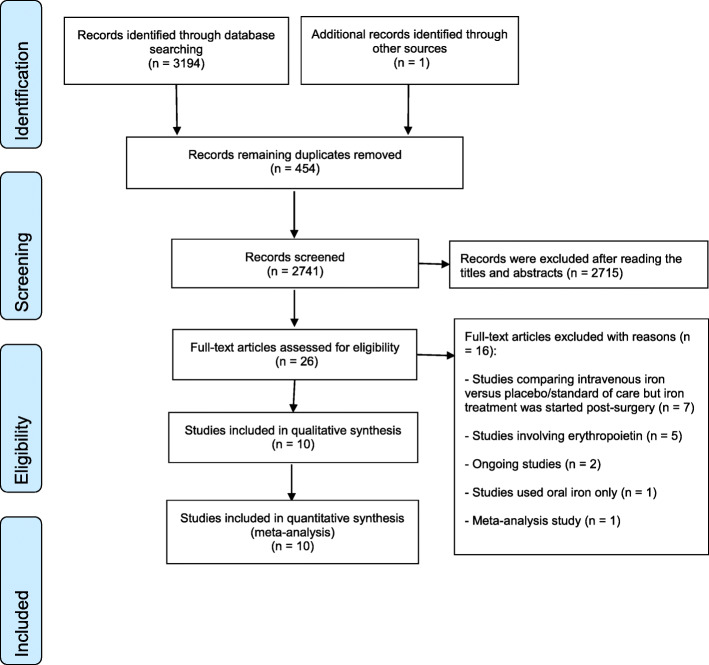
Study flow chart

The initial electronic search yielded 3195 citations. After reviewing the titles and abstracts, twenty-six studies were retrieved for more thorough screening, and a total of ten RCTs met our modified inclusion criteria (Additional File 1 A), as shown in Fig. 1. In these ten studies, there was a total of 1039 participants (530 in the IV iron group and 509 in the control group). All patients were scheduled to have major surgery; two studies involved orthopedic surgery [36, 37], two involved cardiac surgery [38, 39], two involved gynecological surgery [40, 41], two involved colorectal surgery [42, 43], one involved major abdominal surgery [44], and one included a mixture of orthopedic, and cardiovascular surgery [45]. The trial’s characteristics are summarized in Table 1 and Additional File 1 E.

**Table 1:** Summary characteristics of included studies

Study and the publication year	Country	Surgery type	Mean age (SD): intravenous iron/control or oral iron	Patients’ #: intravenous iron/control or oral iron	Anemic patients were recruited	Comparator	Intravenous iron dosage regimen	Last follow-up time
Bernabeu-Wittel et al., 2016 [37]	Spain	Hip fracture surgery	84.6 ± 6.2/82.3 ± 6.9	103/100	Yes	Placebo	1000 mg of IV Ferric carboxymaltose (two 500 mg vials diluted in a bottle of 250 mL of saline after randomization and always previously to surgery.	60 days post-hospital discharge
Edwards et al. 2009 [42]	UK	Colorectal cancer resection	67/70 (median)	34/26	Some patients were anemic	Placebo	300 mg iron sucrose in 2 infusions separated at least 24 h apart, 14 days pre-surgery.	Hospital discharge
Froessler et al. 2016 [44]	Australia	Major abdominal surgery	64 ± 15/68 ± 15	40/32	Yes	Usual care	Ferric carboxymaltose, 15 mg/kg with maximum dose of 1000 mg between 4 and 21 days before surgery. Post-surgery, within 2 days of surgery, participants received 0.5 mg of ferric carboxymaltose per recorded 1 mL of blood loss, if blood loss was > 100 ml.	4 weeks post- surgery.
Garrido-Martín et al. 2012 [38]	Spain	Cardiac surgery	65 ± 11/65 ± 12	54/52	No	Placebo	Three doses of iron sucrose 100 mg/24 h during pre- and postoperative hospitalization.	1 month post-discharge
Johansson et al. 2015 [39]	Denmark	Cardiac surgery	65 ± 8/65 ± 11	30/30	No	Placebo	A single-dose infusion of 1000 mg with a maximum single dose of 20 mg/kg. The injection was a day before surgery or same day.	4 weeks after surgery
Keeler et al. 2017 [43]	UK	Colorectal cancer resection	Median (IQR) 73·8 (67·4–78·6)/74·7 (67·9–80·8)	55/61	Yes	Oral iron	Ferric carboxymaltose with a maximum dose of 1000 mg per week and a maximum of 2000 mg during the trial. The first dose of injection was at least 2 weeks pre-surgery.	2–3 months post-hospital discharge
Kim et al. 2009 [40]	South Korea	Gynecologic surgery for menorrhagia	42.0 ± 7.4/42.3 ± 8.0	30/26	Yes	Oral iron	A 200-mg dose of intravenous iron sucrose three times a week starting 3 weeks prior surgery until target hemoglobin of 10 g/dL was achieved. The treatment started 3 weeks pre-surgery.	Hospital discharge
Serrano-Trenas et al. 2011 [36]	Spain	Hip fracture surgery in elderly patients	83.46 ± 7.1/82.53 ± 6 .4	99/97	Some patients were anemic	Standard protocolized treatment	Iron sucrose 200 mg at 48-hour intervals for 3 doses, starting on the day of admission; the first dose was given pre-surgery. The following doses were administered before or after surgery, depending on the time of surgery.	7 days post-surgery
Shah et al. 2016 [41]	India	Gynecologic surgery for menorrhagia	Most of ages are between 40 and 49 years	55/55	Yes	Oral iron	A 100-mg dose of iron sucrose in 100 ml (2 ampoules) by slow IV infusion. Starting 4-weeks pre-surgery, the dose was repeated on alternate day basis until target hemoglobin of 10 g/dL was achieved.	Hospital discharge
Weisbach et al. 1999 [45]	Germany	Orthopedic or cardio-vascular surgery	64.4 ± 14.7/64.1 ± 9.5	30/30	No	Usual care	A 200-mg dose of iron sucrose, given after each donation and at the enrolment before the first donation.	Hospital discharge

All trials completed the full target dose of IV iron administration preoperatively except for two trials [36, 38] where the injection was initiated preoperatively, and the rest of the dose was completed after surgery. Each trial’s iron injection regimen is detailed in Table 1 and Additional File 1 E.

The follow-up went beyond hospital discharge in four trials [37–39, 44]. The publication dates of the included trials ranged between 1999 [45] and 2017 [43].

### Risk of bias assessment

**Fig. 2:**
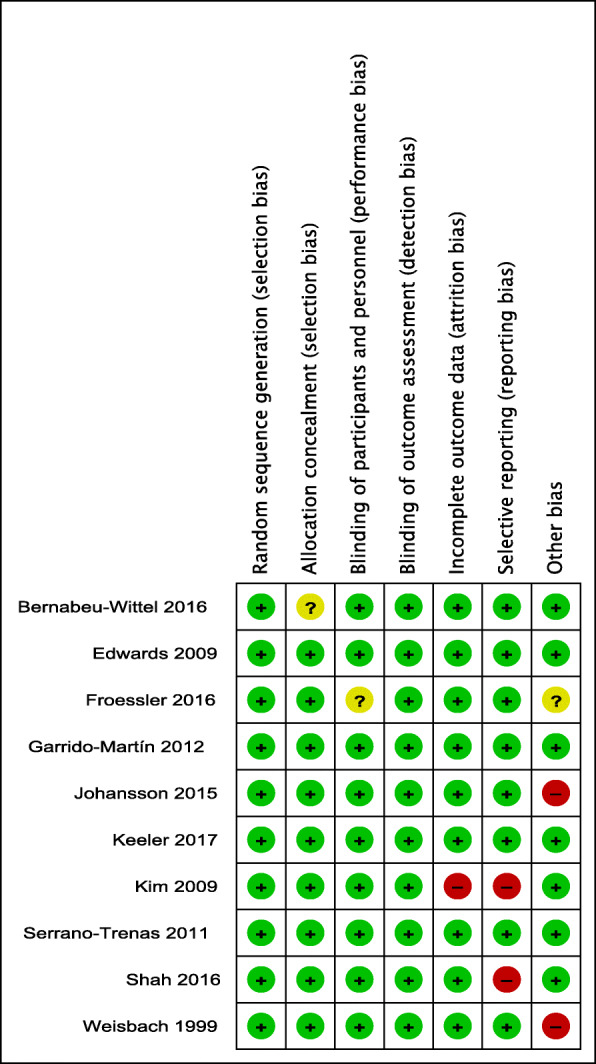
Risk of bias summary shows the authors’ judgment about each risk of bias item for each included study. The symbol “+” represents a low risk of bias, the symbol “−” represents a high risk of bias, and the symbol “?” represents an unclear risk of bias

Four [36, 38, 42, 43] of the ten trials were judged as having a low risk of bias across the entire set of domains, while six trials [37, 39–41, 44, 45] were assessed to have an unclear or high risk of bias in one, or a maximum of two different domains. Details of the risk of bias judgment for each study are shown in Fig. 2, and item-specific judgments for studies are presented in Additional File 1 D.

Except for only one trial [37] that did not provide detailed information on the methods used to achieve allocation concealment, all trials have proper random sequence generation and allocation concealment. Lack of blinding in this kind of study is less likely to generate bias when calculating measurable outcomes like transfusion rate, Hb and ferritin levels, or mortality. Moreover, when comparing IV iron with oral iron, blinding would be difficult due to the effect of oral iron on stool color.

### Exposure for allogeneic blood transfusion

**Fig. 3:**
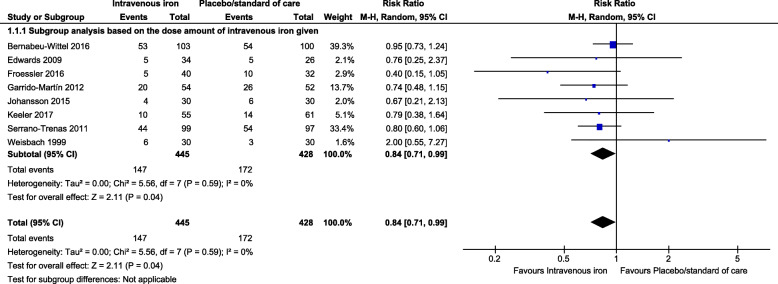
Forest plot comparison shows the pooled comparison effect of intravenous iron therapy versus placebo/standard of care groups on the proportion of the transfused patients (random effects model)

**Fig. 4:**
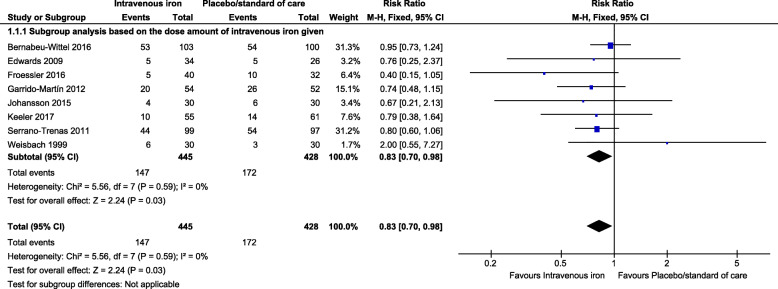
Forest plot comparison shows the pooled comparison effect of intravenous iron therapy versus placebo/standard of care groups on the proportion of the transfused patients (fixed effects model)

Overall, the proportion of transfused patients was 33% in the IV iron versus 40% in the other group. Accordingly, IV iron injection showed significantly higher efficacy, achieving a reduction of 16% in the proportion of patients requiring ABT. This pooled effect estimate was statistically significant (RR between the study groups: 0.84, 95% confidence interval [CI]: 0.71, 0.99, *p* = 0.04) under the random-effects model (Fig. 3). Under the fixed-effects model, the reduction increased slightly to 17% (RR: 0.83, 95% CI: 0.70, 0.98, *p* = 0.03) (Fig. 4). There was no statistical heterogeneity in ABT in either model, with a Cochran *Q* statistic (*p* = 0.59) and a corresponding *I*^2^ statistic of 0%. As a sensitivity analysis, after excluding one study [45] designed mainly to improve autologous blood donation pre-surgery, the reduction of ABT increased slightly to 17% (RR: 0.83, 95% CI: 0.70, 0.98, *p* = 0.02) (Additional File 2 A: Figure 1).

**Fig. 5:**
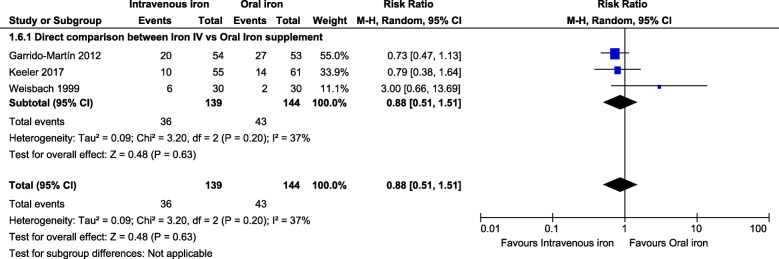
Forest plot comparison shows the pooled direct comparison effect of intravenous iron therapy versus oral iron groups on the proportion of the transfused patients as a subgroup analysis (random effects model)

However, a direct comparison between IV iron versus oral iron trials arms that included only three studies in the analysis was not able to find any transfusion rate difference (Fig. 5).

Given the different kinds of major surgeries using different IV iron preparations with dissimilar injection timing, subgroup analyses were conducted comparing cardiac [38, 39] versus non-cardiac studies [36, 37, 42–44], iron sucrose [36, 38, 42, 45] versus a ferric carboxymaltose IV iron preparation [37, 43, 44], and early injection (> 2 weeks pre-surgery) [43, 45] versus late injection of iron (≤ 2 weeks of surgery) [36–39, 42, 44]. All analyses showed no statistically significant subgroup effect (*p* = 0.52, *p* = 0.98, and *p* = 0.56) respectively (Additional File 2 B: Figure 1-3).

Although we included ten trials, only eight studies [36–39, 42–45] reported transfusion data, so we were not able to conduct a meta-regression [30] or to explore publication bias by checking the funnel plot asymmetry [46] as this requires at least ten trials. Due to Hb measurement timing variability, we did not have ten studies contributing to the same time point analysis.

### Hemoglobin concentration change

**Fig. 6:**
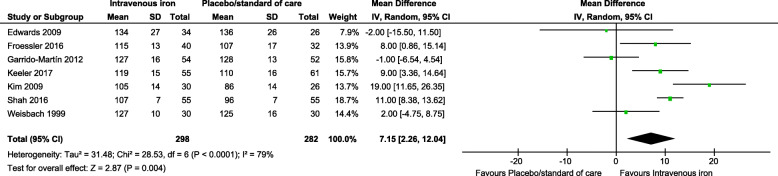
Forest plot comparison shows the pooled comparison effect of intravenous iron therapy versus placebo/standard of care groups on the change of hemoglobin level (g/L) at the post-treatment (pre-surgery) time (random effects model)

**Fig. 7:**
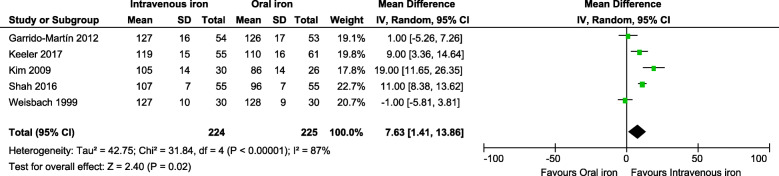
Forest plot comparison shows the pooled comparison effect of intravenous iron therapy versus oral iron groups on the change of hemoglobin level (g/L) at the post-treatment (pre-surgery) time (random effects model)

While the Hb levels MD of baseline-pooled estimate was comparable between groups (Additional File 2 C: Figure 1), after completion of the preoperative IV iron administration, we found a significant Hb level increase in favor of the IV iron group (MD between groups: 7.15 g/L, 95% CI: 2.26, 12.04, *p* = 0.004) (Fig. 6). Similarly, a direct comparison between IV iron versus oral iron participants revealed a significant Hb level rise in favor of the IV iron group (MD between groups: 7.63 g/L, 95% CI: 1.41, 13.86, *p* = 0.02) when the analysis included five studies (Fig. 7). In following this increase difference at pre-surgery, the between-group difference dropped rapidly throughout the hospital stay, and there were no significant differences noted at the first postoperative day or hospital discharge time across groups (Additional File 2 C: Figure 2-3).

**Fig. 8:**
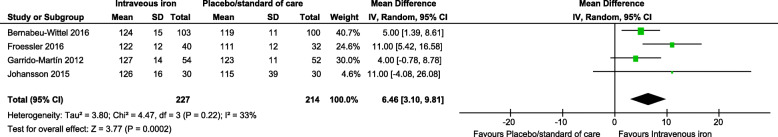
Forest plot comparison shows the pooled comparison effect of intravenous iron therapy versus placebo/standard of care groups on the change of hemoglobin level (g/L) as follow-up > 4 weeks postoperatively (random effects model)

Interestingly, post-hospital discharge, the pooled effect [37–39, 44] showed a significant increase in the Hb difference in favor of the IV iron group (MD between groups: 6.46 g/L, 95% CI: 3.10, 9.81, *p* = 0.0002) (Fig. 8).

### Ferritin levels

While the ferritin levels MD of baseline-pooled estimate was comparable between groups (Additional File 2-D: Figure 1), administration of IV iron was capable of causing marked improvements in the depleted iron stores replenishment in favor of the IV iron group at pre-surgery, hospital discharge, and >  4 weeks postoperatively, (MD between groups: 94.09 ng/mL, 95 % CI: 51.57, 136.61, *p* < 0.0001), (MD between groups: 547.77 ng/mL, 95 % CI: 36.61, 1058.94, *p* = 0.04), and (MD between groups: 347.57 ng/mL, 95 % CI: 290.92, 404.21, *p* < 0.00001) respectively (Additional File 2 D: Figure 2, 4 and 5). In addition, at pre-surgery time, a significant serum ferritin increase was obtained in favor of the IV iron group when it was compared directly with the oral iron group (MD between groups: 106.12 ng/mL, 95 % CI: 32.46, 179.78, *p* = 0.005), (Additional File 2 D: Figure 3).

### Iron-deficiency anemia blood tests

For the rest of the IDA parameters, including reticulocyte (%) percentage, MCV, hematocrit value (%), transferrin saturation value (TSAT %), MCH, MCHC, and serum iron level, only a few trials contributed to the different analyses (Additional File 2 D: Figure 6-24). In favor of the IV iron group, there are substantial increases in the reticulocyte percentage (at both pre-surgery time and hospital discharge time); and in the mean MCV level (at pre-surgery time). For the rest of these parameters, there was no significant change across groups after iron injection.

### Adverse events

The participants tolerated IV iron injection well, and for non-serious (mild or moderate) AEs, there was no difference across groups (RR: 1.13, 95% CI: 0.78, 1.65, p = 0.52 (Additional File 2 E: Figure 1). After including a trial of zero events [38] , the results remained similar [36–38, 40, 41, 43–45]. Regarding serious adverse effects (SAEs), similarly, there was no difference between groups (RR: 0.96, 95% CI: 0.44, 2.10, *p* = 0.92) [39, 43] (Additional File 2 E: Figure 2).

### Mortality

There was no statistically significant difference between the two groups for the reported 30-day mortality (RR: 1.10, 95% CI: 0.60, 2.00, *p* = 0.76) [36, 37, 39, 44] (Additional File 2 E: Figure 3). Findings were alike after including a trial of zero events [39]. At 2 months post-hospital discharge, comparable death results (RR: 1.18, 95% CI: 0.63, 2.19, *p* = 0.6) were also obtained [37, 43] (Additional File 2 E: Figure 4).

### Infection

Five trials [36–38, 43, 44] reported data for postoperative infection. One of these five studies [38] reported a similar infection rate between the trial arms but did not provide numbers. Another study [36] reported the total infection rate across the study (14.8%) but did not provide arm-specific numbers of infections. A third study [43] reported no difference in infection rate (*p* = 0.091) or grade of its severity (*p* = 0.083) between the trial arms but did not provide numbers. The other two studies [37, 44] showed similar infection incidence between groups (RR: 0.64, 95% CI: 0.30, 1.40, *p* = 0.27) (Additional File 2 E: Figure 5).

### Hospital length of stay

Five trials [36, 37, 42–44] reported data about hospital LOS. However, none of the individual studies showed a statistical difference across the trial arms, and we were not able to pool them as four trials [37, 42–44] provided the LOS as median days.

### Quality of life

Health-related quality of life data were provided in only two trials [37, 44] that used two different tests, which makes quantitative analysis not feasible. The first trial [37] used the Short Form 36 Version 2 for acute patients (SF-36v2) [47], at 60 days post-hospital discharge reassessment; it revealed statistically non-significant and clinically unimportant changes. Similar findings were observed in the second trial [44] that used the short form health survey (SF36) [48] at 4 weeks post-surgery reassessment.

## Discussion

The core finding of this meta-analysis, based on moderate-quality evidence, was a 16% reduction in the ABT for the participants who received the iron injection (Additional File 3). In addition, pre-surgery Hb and ferritin levels increase accompanied by ABT reduction, with no change in postoperative infections, AE, or mortality rate.

For transfusion rate reduction, the minimal clinically significant difference (MCID) cutoff has not been established yet. However, the largest currently ongoing trial investigating iron injection to treat preoperative anemia in major surgery used a risk reduction of 12 % in the transfusion rate to calculate the sample size [49]. Therefore, in this meta-analysis, it would seem that after receiving IV iron, a reduction of 16% in the proportion of patients requiring ABT might be considered clinically important and sufficient to change patient management. This transfusion reduction in our study is in accordance with previous large meta-analysis of both surgical and nonsurgical RCTs [50], a recent surgical meta-analysis [51], several observational studies [52–61], and a pooled analysis of observational data from 2547 patients [62]. This finding is of great benefit for all patients, including Jehovah’s Witness (JW) patients, where ABT is forbidden, patients who will be potentially organ recipients, patients for whom transfusions are medically contra-indicated, and those who are living in countries with restricted resources.

In the current meta-analysis, we included trials from different surgical specialties that allow for extended external validity; however, these findings may not be generalizable to patient populations other than those undergoing elective surgery. In particular, the findings from this meta-analysis conflict with a recent trial in critical care patients [63], and a meta-analysis in pregnancy [20]. In these two studies, although the Hb levels were significantly higher after IV iron therapy, there were no significant differences in blood transfusion between groups [20, 63]. Moreover, a recent critical care meta-analysis [64] reported no difference in ABT requirements or Hb level change at short-term (up to 10 days) or mid-term follow-up between groups after IV iron treatment. In the latter meta-analysis, findings were limited by considerable heterogeneity between trials, including the nature of interventions and the risks of bias in the included studies. More recently, in surgical settings, two meta-analyses did not show evidence of transfusion reduction in non-elective surgery [65], or when iron injected postoperatively [66], indicating the importance of injecting iron pre-surgery by sufficient time.

In our meta-analysis, although there was no statistical heterogeneity as *I*^2^ was zero% in both random and fixed-effects models, there was a discrepancy in ABT reduction rate between the two models that could ensue due to type I error indicating a shortage of power to identify trials' heterogeneity. However, the clinical heterogeneity of trials was explored using sensitivity and subgroup analyses. Our subgroup analyses were conducted by comparing cardiac [38, 39] versus non-cardiac surgeries [36, 37, 42–44], iron sucrose [36, 38, 42, 45] versus carboxymaltose IV iron preparation [37, 43, 44], and early [43, 45] versus delayed iron injection [36–39, 42, 44]. Interestingly, none of these analyses showed any statistically significant subgroup influence to modify the effect of IV iron. We think that this analysis may not be able to identify subgroup differences due to a smaller number of studies/participants and/or uneven covariate distribution, producing uncertainty in the results.

Interestingly, in our meta-analysis, the Hb increase tendency had a bi-phasic pattern composed of two waves. This pattern rise might be related to the IV iron timing variability of administration either early (2–5 weeks before the operation) in some trials [40–43, 45] or late (around surgery time) in other trials [36–39, 44]. The disappearance of the first wave might be related to the attenuation/termination of the IV iron effect and/or the substantial perioperative acute blood loss. One study [44] that administered about a thousand grams of the IV iron 4–21 days pre-surgery in addition to extra doses of IV iron increments postoperatively contributed to a positive effect in the pooled estimate in both Hb rise waves indicating the importance of the early and second dose of IV iron injection. Overall, this detected Hb increase in our meta-analysis is consistent with two feasibility studies [67, 68], and several observational studies [53, 56, 69–71], where IV iron was given pre-surgery. Moreover, our Hb increase pattern over time is in line with a very recent surgical meta-analysis where IV iron was capable of augmenting Hb level. This increase was higher when it was measured at 2 to 4 weeks of follow-up (MD = 5.69 g/L, 95% CI: 2.11–9.27), but not when calculated earlier [66] supporting a previous surgical trial that showed IV iron achieved an increase at 4 weeks after IV iron administration [72]. These findings, and ours, suggest that iron injection should be initiated about 4 weeks pre-surgery and continued till surgery using a second dose with at least a week interval from the first injection in order to achieve a long and sustained effect to stimulate the erythropoiesis on time to protect against ABT through moving the peak effect to be at the perioperative time [53, 70].

The other observation was that the Hb rise did not happen when the trial’s participants received a total dose of ≤ 600 mg of IV iron [36, 38, 42], only the rise occurred significantly when a higher dose of iron was injected [37, 39–41, 43, 44], demonstrating a pattern of positive dose-response relationship supporting the recent consensus recommendation to give a dose between 1000 and 1500 mg of IV iron in one or two divided doses [73]. As our meta-analysis is for the RCTs, it would be plausible to infer a causal relationship between iron injection and Hb increase tendency. Accordingly, we think that patients who receive a total dose of < 1000 mg of iron or who are injected only around the time of surgery would not respond as well as those receive larger dose administered at least 2 weeks pre-surgery.

In our meta-analysis, although the Hb rise by 7.15 g/L is statistically significant, this rise is still considered below the defined clinical meaningful hemoglobin-concentration increase (≥1 g/dL) [74]. Interestingly, this modest Hb increase was associated with meaningful transfusion reduction.

Another observation is that the ferritin rise peak was at hospital discharge time preceding the second wave of the Hb rise as iron replenishment is a process that happens before Hb formation. These data support other investigators’ findings [53, 70]. Another finding we found was the reticulocyte percentage/count increase post-treatment and during the hospital stay, demonstrating a continued recent bone marrow activity to prepare for the second wave appearance.

We did not find a significant difference in AEs occurrence between the groups, although six trials [37, 39–41, 43, 44] injected doses of ≥ 1000 to 2000 mg of iron in one trial [43]. This finding is in harmony with nonsurgical trials using up to 2000 mg [75] or 2500 mg of IV iron [76], two surgical meta-analyses [65, 77], a meta-analysis in IDA [78], and surgical observational studies [56, 79]. The chief AEs related to IV iron administration in our study were erythema with pain/itching at the injection site, phlebitis, discomfort, fever, and dizziness. Nausea, vomiting, constipation, and diarrhea were more frequent in those participants who received oral iron.

Given the relatively small sample size of each trial and the relative rarity of severe side effects as the anaphylactic shock reactions, there is a need for a large-scale phase III clinical trial or a large observational report to study the incidence of the AEs of IV iron. However, according to the United States Death Certificate Registry, only three mortalities were coded as “AEs in therapeutic use of iron preparation” over the period between 1979 and 2006 [80], and the actual anaphylaxis was very rare [16].

In this meta-analysis, the iron injection did not change our study mortality, LOS, or postoperative infection rates, consistent with a recent surgical meta-analysis [65].

### Strengths of the study

This meta-analysis protocol was registered and published. We followed the recommendations of the Cochrane collaboration and PRISMA statement. Our search was comprehensive without having any time, language, or IV iron preparation restrictions. It has broad external generalizability for many surgical subspecialties. As it included only RCTs, it provides high-quality evidence in practice. All analyses were performed using both fixed-effect and random-effect models. When in between-study heterogeneity exists, both techniques may be biased, but random-effects models will be more conservative.

### Limitations of the study

The first limitation is some of the included studies recruited non-anemic patients, which would bias their findings toward the null. Second, the current study findings may not be generalizable to younger patients undergoing major surgery as eight of the ten trials had a mean/median age above 64 years old. Only two gynecological studies investigated younger female patients experiencing menorrhagia; a condition that usually exists at a younger age. A third potential limitation of this meta-analysis is the relatively smaller sample size of most of the trials that necessitate careful interpretation of the reported side effects of IV iron since more extensive studies are needed to identify side effects. The fourth limitation is that we estimated/imputed SD for trials when these were not published. This could be a source of bias, although the alternative would have been to exclude those trials. Doing so would have resulted in a smaller sample size of this meta-analysis and would have introduced another kind of bias that would have made the interpretation of findings difficult.

## Conclusion

Based on moderate-quality evidence, our results are in support of preoperative intravenous iron administration to reduce the likelihood of allogeneic blood transfusion and to provide a modest increase in hemoglobin concentration in major surgery settings. This evidence came from eight trials for the transfusion proportion and seven trials for the hemoglobin change.

Similarly, with moderate-quality evidence, a greater increase in serum ferritin pre-surgery was found post iron injection.

Low-quality evidence suggests that surgical patients tolerate the non-serious adverse events of intravenous iron supplementation. However, with high-quality evidence, no evidence suggests that iron injection decreases mortality.

However, our findings did not show the superiority of parenteral iron therapy over oral iron on the proportion reduction of the transfused patients as a subgroup analysis. Accordingly, new trials could be made to have a robust evidence-body, and we recommend a robust clinical trial testing the hypothesis that an optimally timed injected iron in a large dose (1500–2000 mg) is safe to counteract acute perioperative blood loss.

## Supplementary Information

**Additional file 1.** A: Modified Article Selection Criteria. B: PRISMA Checklist. C: Search Strategy. D: Risk of Bias Assessment. E: Characteristics of Included Studies’ Tables.

**Additional file 2.** A: Forest-plots for the Sensitivity Analysis. B: Forest-plots for the Subgroup Analysis. C: Forest-plots for Hemoglobin Values at Different Time Points. D: Forest-plots for the Different Iron-deficiency Anemia Blood Tests. E: Forest-plots for the Safety Endpoints of Intravenous Iron versus Placebo or Oral Iron.

**Additional file 3.** Summary of Findings Table.

## Data Availability

Study protocol: available at www.crd.york.ac.uk/prospero (PROSPERO: CRD42018098604).

## Abbreviations

ABT: Allogeneic blood transfusion
IDA: Iron-deficiency anemia
IV: Intravenous
Hb: Hemoglobin
RCT: Randomized controlled trial
AEs: Adverse events
PROSPERO: Prospective Register of Systematic Reviews
PRISMA: Preferred Reporting Items for Systematic Reviews and Meta-analyses
CPCI: Conference Proceedings Citation Index
RR: Risk ratios
CIs: Confidence intervals
MD: Mean difference
SD: Standard deviation
MCV: Mean corpuscular volume
TSAT%: Transferrin saturation value
MCH: Mean corpuscular hemoglobin
MCHC: Mean corpuscular hemoglobin concentration
SAEs: Serious adverse effects
LOS: Hospital length of stay
HRQoL: Health-related quality of life
SF-36v2: Short form 36 version 2 for acute patients
SF36: Short form health survey
MCID: The minimal clinically important difference
JW: Jehovah Witness
