# Supplementary material for: Role of preoperative intravenous iron therapy to correct anemia before major surgery: a systematic review and meta-analysis
Source: Syst Rev. 2021 Jan 23;10:36. doi: 10.1186/s13643-021-01579-8 (PMC7824930; doi:10.1186/s13643-021-01579-8)
Supplement: Supplementary file 1 — Additional file 1. A: Modified Article Selection Criteria. B: PRISMA Checklist. C: Search Strategy. D: Risk of Bias Assessment. E: Characteristics of Included Studies’ Tables. [file 13643_2021_1579_MOESM1_ESM.docx]

ADDITIONAL FILE 1

**Additional File 1-A: Modified Article Selection Criteria**

Inclusion criteria

Eligibility criteria of studies were as follows:

- Designs included were randomized and quasi-randomized studies in all different phases of clinical research.
- Study compared any intravenous (IV) iron preparation that was initiated preoperatively to placebo or standard of care (oral iron).
- Study reported findings specific to adult humans.
- Study included patients undergoing any elective major surgery including, but not limited to, cardiac, vascular, thoracic, orthopedic, gastrointestinal, brain, urologic, or gynecologic operations.
- Study reported at least one of the two following outcomes: absolute or relative change in preoperative hemoglobin (Hb) concentration and/or the proportion of patients received perioperative allogenic blood transfusion (ABT).

Exclusion criteria

Anyone of these criteria resulted in a study being excluded:

- Observational (non-experimental) studies, reviews, opinion papers, letters to the editor, and studies with no reported methodology.
- Studies in pediatric patients.
- Studies used oral iron only.
- Studies used IV iron plus erythropoietin (EPO).
- Studies of minimally invasive robotic and laparoscopic surgery.

**Additional File 1-B: PRISMA Checklist**

| **Section/topic** | **#** | **Checklist item** | **Reported on page #** |
| --- | --- | --- | --- |
| **TITLE** | | |  |
| Title | 1 | Identify the report as a systematic review, meta-analysis, or both. | p. 1 |
| **ABSTRACT** | | |  |
| Structured summary | 2 | Provide a structured summary including, as applicable: background; objectives; data sources; study eligibility criteria, participants, and interventions; study appraisal and synthesis methods; results; limitations; conclusions and implications of key findings; systematic review registration number. | p. 1-2 |
| **INTRODUCTION** | | |  |
| Rationale | 3 | Describe the rationale for the review in the context of what is already known. | p. 3 |
| Objectives | 4 | Provide an explicit statement of questions being addressed with reference to participants, interventions, comparisons, outcomes, and study design (PICOS). | p. 3 |
| **METHODS** | | |  |
| Protocol and registration | 5 | Indicate if a review protocol exists, if and where it can be accessed (e.g., Web address), and, if available, provide registration information including registration number. | p. 5 |
| Eligibility criteria | 6 | Specify study characteristics (e.g., PICOS, length of follow-up) and report characteristics (e.g., years considered, language, publication status) used as criteria for eligibility, giving rationale. | p. 6 |
| Information sources | 7 | Describe all information sources (e.g., databases with dates of coverage, contact with study authors to identify additional studies) in the search and date last searched. | p. 6-7 & Additional File 5 |
| Search | 8 | Present full electronic search strategy for at least one database, including any limits used, such that it could be repeated. | Additional file 3 |
| Study selection | 9 | State the process for selecting studies (i.e., screening, eligibility, included in systematic review, and, if applicable, included in the meta-analysis). | p. 7 |
| Data collection process | 10 | Describe method of data extraction from reports (e.g., piloted forms, independently, in duplicate) and any processes for obtaining and confirming data from investigators. | p. 8 |
| Data items | 11 | List and define all variables for which data were sought (e.g., PICOS, funding sources) and any assumptions and simplifications made. | p. 7 |
| Risk of bias in individual studies | 12 | Describe methods used for assessing risk of bias of individual studies (including specification of whether this was done at the study or outcome level), and how this information is to be used in any data synthesis. | Additional file 4 |
| Summary measures | 13 | State the principal summary measures (e.g., risk ratio, difference in means). | p. 8-9 |
| Synthesis of results | 14 | Describe the methods of handling data and combining results of studies, if done, including measures of consistency (e.g., I^2^) for each meta-analysis. | p. 8 |
| **Section/topic** | **#** | **Checklist item** | **Reported on page #** |
| Risk of bias across studies | 15 | Specify any assessment of risk of bias that may affect the cumulative evidence (e.g., publication bias, selective reporting within studies). | p. 10 & Additional file 5 |
| Additional analyses | 16 | Describe methods of additional analyses (e.g., sensitivity or subgroup analyses, meta-regression), if done, indicating which were pre-specified. | p. 9 |
| **RESULTS** | | |  |
| Study selection | 17 | Give numbers of studies screened, assessed for eligibility, and included in the review, with reasons for exclusions at each stage, ideally with a flow diagram. | p. 9, Figure 1 |
| Study characteristics | 18 | For each study, present characteristics for which data were extracted (e.g., study size, PICOS, follow-up period) and provide the citations. | page 9  Table 1 and Additional File 5 |
| Risk of bias within studies | 19 | Present data on risk of bias of each study and, if available, any outcome level assessment (see item 12). | Page 10, Figure 2, Additional files 4 and 5 |
| Results of individual studies | 20 | For all outcomes considered (benefits or harms), present, for each study: (a) simple summary data for each intervention group (b) effect estimates and confidence intervals, ideally with a forest plot. | Figure 3,4 and 5  Additional files 10 and 11 |
| Synthesis of results | 21 | Present results of each meta-analysis done, including confidence intervals and measures of consistency. | Additional file 11 |
| Risk of bias across studies | 22 | Present results of any assessment of risk of bias across studies (see Item 15). | Figure 2 and Additional files 4 and 5. |
| Additional analysis | 23 | Give results of additional analyses, if done (e.g., sensitivity or subgroup analyses, meta-regression [see Item 16]). | p. 8-9, Additional file 6,  and Additional file 7 |
| **DISCUSSION** | | |  |
| Summary of evidence | 24 | Summarize the main findings including the strength of evidence for each main outcome; consider their relevance to key groups (e.g., healthcare providers, users, and policy makers). | p. 15-19  Additional file 11 |
| Limitations | 25 | Discuss limitations at study and outcome level (e.g., risk of bias), and at review-level (e.g., incomplete retrieval of identified research, reporting bias). | p. 19-20 |
| Conclusions | 26 | Provide a general interpretation of the results in the context of other evidence, and implications for future research. | p. 15-17 |
| **FUNDING** | | |  |
| Funding | 27 | Describe sources of funding for the systematic review and other support (e.g., supply of data); role of funders for the systematic review. | p. 30 |

*From:*  Moher D, Liberati A, Tetzlaff J, Altman DG, The PRISMA Group (2009). Preferred Reporting Items for Systematic Reviews and Meta-Analyses: The PRISMA Statement. PLoS Med 6(7): e1000097. doi:10.1371/journal.pmed1000097

**Additional File 1-C: SAMPLE SEARCH STRATEGY**

*MEDLINE through OVID search strategy*

Database: Ovid MEDLINE(R) In-Process & Other Non-Indexed Citations, Ovid MEDLINE(R) Daily and Ovid MEDLINE(R) <1946 to Present> February 01, 2019

1. Iron/

2. exp Iron Compounds/

3. (iron or dextran or venofer or ferric or ferrous or ferrlecit).mp. [mp =title, abstract, original title, name of substance word, subject heading word, keyword heading word, protocol supplementary concept, rare disease supplementary concept, unique identifier]

4. 1 or 2 or 3

5. exp Anemia/

6. (anemi* or anaemi*).mp. [mp =title, abstract, original title, name of substance word, subject heading word, keyword heading word, protocol supplementary concept, rare disease supplementary concept, unique identifier]

7. 5 or 6

8. 4 and 7

9. exp perioperative care/ or exp perioperative period/

10. exp Specialties, Surgical/

11. exp Surgical Procedures, Operative/

12. (preoperat* or postoperat* or perioperat* or operati* or surg* or presurg* or postsurg* or perisurg*).mp. [mp =title, abstract, original title, name of substance word, subject heading word, keyword heading word, protocol supplementary concept, rare disease supplementary concept, unique identifier]

13. 9 or 10 or 11 or 12

14. 8 and 13

15. randomized controlled trial.pt.

16. clinical trial.pt.

17. randomi?ed.ti,ab.

18. placebo.ti,ab.

19. dt.fs.

20. randomly.ti,ab.

21. trial.ti,ab.

22. groups.ti,ab.

23. or/15-22

24. animals/

25. humans/

26. 24 not (24 and 25)

27. 23 not 26

28. 14 and 27

**Additional File 1-D: Risk of Bias Assessment**

Risk of bias graph shows the authors' judgment about each risk of bias item presented as percentages across all included studies.

**Additional File 1-E: Characteristics of Included Studies’ Tables**

**Table 1: Bernabeu-Wittel et al., 2016**

| ***Methods*** | ***Participants characteristics*** | |
| --- | --- | --- |
| Country and year | Spain 2016 | |
| Type of surgery | Hip fracture surgery | |
| Participants number: Intravenous iron/Placebo | 103/100 | |
| Post-randomization dropout(s): | Zero patients. However, 3/306 were excluded before randomization because of not meeting the inclusion criteria (1 patient had periprothesic fracture and 2 patients were receiving oral anticoagulation | |
| Main age (y): Intravenous iron/ Placebo | 84.6 ± 6.2/82.3 ± 6.9 | |
| Female sex (%)  Intravenous iron: Placebo | 82: 87 | |
| Control arm: | Placebo | |
| Inclusion criteria: | Patients of age at least 65 years, with osteoporotic hip fracture requiring surgical repair, hemoglobin levels between 90 and 120 g/L, and signed informed trial participation consent form. | |
| Exclusion criteria: | Patients with bone marrow diseases that could interfere in the erythropoietic process, blood coagulation diseases or current treatment with anticoagulants, documented allergy or intolerance and/or contraindication to EPO use and/or IV iron, rheumatoid arthritis and/or another demonstrated origin of inflammatory anemia and/or uncontrolled arterial hypertension, current or previous treatment with EPO or IV iron for at least 3 months, and chronic renal failure receiving hemodialysis or peritoneal dialysis. | |
| Intervention | Participants were randomly allocated to one of the following groups:  Group 1: IV iron arm who received 1000 mg of IV FCM (two 500 mg vials diluted in a bottle of 250 mL of saline, with opaque plastic bag and infusion system), in a 20-minute infusion after randomization and always before surgery.  Group 2: placebo arm who received subcutaneous single-dose placebo (saline) in a prefilled syringe IV placebo (250 mL of saline, with opaque plastic bag and infusion system), in a 20-min infusion. | |
| Trial’s outcomes reported and related to our primary outcomes | Outcomes reported were change in hemoglobin levels and blood transfusion requirements. | |
| Time of outcomes measurements | Hemoglobin levels were measured at 24 and 72 hours after surgery, at discharge, and after 60 days of the discharge. Transfusion requirement was measured during hospitalization and up to 60 days of hospital discharge. | |
| Last follow up time | Sixty days of hospital discharge | |
| Additional notes | The trial recruited anemic patients  Attempts to contact the trial authors to request more data were unsuccessful. | |
| ***Risk of bias assessment*** | | |
| Risk of bias item | Authors’ judgement | Authors’ backing for judgement |
| Random sequence generation  (selection bias) | Low risk | Quote: “The randomization assignment list was stratified by centers and performed by unequal blocks technique.” |
| Allocation concealment  (selection bias) | Unclear risk | Details not provided |
| Blinding of participants and researchers (performance bias) | Low risk | Comment: blinding of the participants and researchers using opaque infusion sleeves. |
| Blinding of outcome assessment  (detection bias) | Low risk | Comment: not stated. However, primary outcomes as transfusion rate and hemoglobin level measurement are objective, which are unlikely to be Influenced. |
| Incomplete outcome data  (attrition bias) | Low risk | Comment: reasons for missing data were reported and balanced across both trial groups. |
| Selective reporting  (reporting bias) | Low risk | Comment: Important clinical outcomes including blood transfusion rate, and hemoglobin level changes were reported. |
| Other potential source of bias | Low risk | Comment: no authors’ financial conflict of interest recognised. |

**Table 2: Edwards et al. 2009**

| ***Methods*** | ***Participants characteristics*** | |
| --- | --- | --- |
| Country and year | UK 2009 | |
| Type of surgery | Colorectal cancer resection | |
| Participants number: Intravenous iron/Placebo | 34/26 | |
| Post-randomization dropout(s): | One patient from each arm discontinued the intervention and failed to attend for the second infusion. | |
| Main age (y): Intravenous iron/ Placebo | 67/70 (median) | |
| Female sex (%)  Intravenous iron: Placebo | 35: 35 | |
| Control arm: | Placebo | |
| Inclusion criteria: | Adult patients undergoing bowel resection surgery for colorectal cancer. | |
| Exclusion criteria: | Participants who were under 18-years old, received blood transfusion, or oral iron in the last six weeks of the day they were approached. Also, the patients who is going to have their operation within 15 days of date of recruitment. | |
| Intervention | Participants were randomly allocated to one of the following groups:  Group 1: IV iron arm who received 300 mg iron sucrose in 2 infusions separated at least 24h apart, 14 days pre-surgery.  Group 2: placebo arm who received an equal volume of normal saline. | |
| Trial’s outcomes reported and related to our primary outcomes | Outcomes reported were change in hemoglobin levels and blood transfusion requirements. | |
| Time of outcomes measurements | Hemoglobin levels were measured at recruitment, pre-surgery surgery, post-surgery, and at hospital discharge. Transfusion requirement was measured perioperatively. | |
| Last follow up time | Hospital discharge | |
| Additional notes | The trial recruited anemic and non-anemic patients.  Attempts to contact the trial authors to request more data were unsuccessful. | |
| ***Risk of bias assessment*** | | |
| Risk of bias item | Authors’ judgement | Authors’ backing for judgement |
| Random sequence generation  (selection bias) | Low risk | Quote: “Participants were allocated to either the treatment (iron) group or a placebo group, based on a computer-generated randomization sequence provided by the Research and Development Support Unit. To ensure equal numbers of anemic patients in each treatment group, randomization was stratified according to prerecruitment Hb status: normal (Hb level at least 13·5 g/dl in males and 12·5 g/dl in females), anemic, or unknown (no test within 2 months of recruitment). Block randomization was used to ensure similar numbers in each group for each subset.” |
| Allocation concealment  (selection bias) | Low risk | Quote: “Allocation codes were sealed in sequentially numbered opaque envelopes which were secured within a locked store room in a dedicated research unit” |
| Blinding of participants and researchers (performance bias) | Low risk | Quote: “Although the investigator administering the infusion fusion was not blinded to the treatment group, this was concealed from the patient by using an opaque sheath to cover the drug giving set. The chief investigator and clinicians involved in perioperative care also remained blinded to the treatment group for the duration of the trial” |
| Blinding of outcome assessment  (detection bias) | Low risk | Quote: “The chief investigator and clinicians involved in perioperative care also remained blinded to the treatment group for the duration of the trial.”  Comment: hemoglobin measurement and transfusion rate as objective outcome measurement are unlikely to be influenced. |
| Incomplete outcome data  (attrition bias) | Low risk | Comment: two post-randomization dropouts were reported (one in each arm) and there was no loss to follow-up. |
| Selective reporting  (reporting bias) | Low risk | Comment: Important clinical outcomes including blood transfusion rate, and hemoglobin level changes were reported. |
| Other potential source of bias | Low risk | Comment: no authors’ financial conflict of interest recognised. |

**Table 3: Froessler et al. 2016**

| ***Methods*** | ***Participants characteristics*** | |
| --- | --- | --- |
| Country and year | Australia 2016 | |
| Type of surgery | Major Abdominal Surgery | |
| Participants number: Intravenous iron/Usual care | 40/32 | |
| Post-randomization dropout(s): | not stated | |
| Main age (y): Intravenous iron/ Usual care | 64 ± 15/68 ±15 | |
| Female sex (%)  Intravenous iron: Usual care | 53: 47 | |
| Control arm: | Usual care | |
| Inclusion criteria: | Adult patients who were >18 years with ferritin <300 mcg/L, transferrin saturation <25%, Hb <12.0 g/dL for women, Hb <13.0 g/dL for men. | |
| Exclusion criteria: | Patients under 18-years old. | |
| Intervention | Participants were randomly allocated to one of the following groups:  Group 1: IV iron arm who received ferric carboxymaltose, given as a single dose over 15 minutes, before surgery (simplified dosing protocol; 15mg/kg bodyweight to a maximum dose of 1000 mg). Postoperatively, within two days of surgery, participants received 0.5 mg of ferric carboxymaltose per recorded 1 mL of blood loss, if blood loss was at least 100 ml.  Group 2: placebo arm who received usual care. | |
| Trial’s outcomes reported and related to our primary outcomes | Outcomes reported were change in hemoglobin levels and blood transfusion requirements. | |
| Time of outcomes measurements | Hemoglobin levels were measured at recruitment, pre-surgery, post-surgery, at discharge, and 4 weeks post-surgery. Transfusion requirement was measured in the 21 days pre-surgery till post-surgery. | |
| Last follow up time | Four weeks post-surgery | |
| Additional notes | The trial recruited anemic patients only.  Attempts to contact the trial authors to request more data were unsuccessful. | |
| ***Risk of bias assessment*** | | |
| Risk of bias item | Authors’ judgement | Authors’ backing for judgement |
| Random sequence generation  (selection bias) | Low risk | Quote: “Randomization followed a computer-generated number of sequence and allocation was conducted by telephone” |
| Allocation concealment  (selection bias) | Low risk | Quote: “The surgeon performing the operation was informed of patient participation in the study but group allocation was not revealed” |
| Blinding of participants and researchers (performance bias) | unclear risk | Quote: “The surgeon performing the operation was informed of patient participation in the study but group allocation was not revealed”  Comment: there was no blinding of participants mentioned. This was unclear if this might impact blood transfusion administration, or the quality of life scores. Also, no placebo administered to the usual care group. |
| Blinding of outcome assessment  (detection bias) | Low risk | Quote: “This was performed by an independent statistician on the interim data-monitoring committee with the data blinded (intervention group n = 32, usual care group n = 26)” |
| Incomplete outcome data  (attrition bias) | Low risk | “the study was terminated early due to higher than expected rates of poor outcome in the usual care group. At the time of study termination, 72 eligible patients were enrolled and randomized (intervention group n = 40, usual care group n = 32)” |
| Selective reporting  (reporting bias) | Low risk | Comment: Important clinical outcomes including blood transfusion rate, and hemoglobin level changes were reported. |
| Other potential source of bias | unclear risk | Comment: the trial was terminated early after interim data analysis showed high than anticipated rates of red blood cell transfusion in the usual care arm |

**Table 4: Garrido-Martín et al. 2012**

| ***Methods*** | ***Participants characteristics*** | |
| --- | --- | --- |
| Country and year | Spain 2012 | |
| Type of surgery | Cardiac surgery | |
| Participants number: Intravenous iron/Placebo | 54/52 | |
| Post-randomization dropout(s): | In the IV iron group, five patients did not receive allocated intervention, ten patients discontinued the intervention, and two patients were lost to follow-up. In the placebo group, three patients did not receive allocated intervention, seven patients discontinued the intervention, three patients were lost to follow-up and one patient excluded from the analysis due to lack of data to analyze. | |
| Main age (y): Intravenous iron/ Placebo | 65 ± 11/65 ± 12 | |
| Female sex (%)  Intravenous iron: Placebo | 30: 23 | |
| Control arm: | Placebo | |
| Inclusion criteria: | Patients older than 18 years of age, elective on-pump cardiac surgery, without previous anemia, susceptible to treatment, without preoperative blood transfusion, able to complete all study visits and providing written informed consent. | |
| Exclusion criteria: | Elective off-pump cardiac surgery patients, patient who received fibrinolytic therapy 48 hours before on-pump surgery, history of impaired renal function (creatinine clearance <50 ml/min), previous surgery for active endocarditis, redo-surgery patients, pregnant or lactating, signs of active gastrointestinal bleeding, vitamin B12 deficit, ferropenic anemia, clinical history of asthma or allergy, active infection, included in another clinical study, hepatic disease, history of allergy to iron, unlikely to adhere to protocol follow-up, unable to comply with the study protocol. | |
| Intervention | Participants were randomly allocated to one of the following groups:  Group 1: IV iron arm who received (III)-hydroxide sucrose complex (Venofer®; Uriach Lab.) for three doses of 100 mg of intravenous iron/24 h during pre- and postoperative hospitalization,  Group 2: placebo arm who received oral and intravenous and oral placebo pre- and postoperatively. | |
| Trial’s outcomes reported and related to our primary outcomes | Outcomes reported were change in hemoglobin levels and blood transfusion requirements. | |
| Time of outcomes measurements | Hemoglobin levels were measured at entry operating room exit operating room, ICU admission, ICU discharge, at hospital discharge and at a month of discharge. Transfusion requirement was measured in the operating room, in the ICU, and in the cardiac ward. | |
| Last follow up time | One month after hospital discharge | |
| Additional notes | The trial recruited non-anemic patients.  Attempts to contact the trial authors to request more data were unsuccessful. | |
| ***Risk of bias assessment*** | | |
| Risk of bias item | Authors’ judgement | Authors’ backing for judgement |
| Random sequence generation  (selection bias) | Low risk | Quote: “Randomization was performed by the Hospital Research Unit using a computer-generated random numbers list.” |
| Allocation concealment  (selection bias) | Low risk | Quote: “Randomization was performed by the Hospital Research Unit using a computer-generated random numbers list.” |
| Blinding of participants and researchers (performance bias) | Low risk | Quote: “All medication, including placebo, was prepared by the Department of Pharmacy and assigned to patients according to the random numbers list, so neither the medical staff nor the patients knew what treatment was being used. All intravenous solutions were presented in disguised form” |
| Blinding of outcome assessment  (detection bias) | Low risk | Comment: not stated, however, hemoglobin measurement and transfusion rate as objective outcome measurement are unlikely to be influenced. |
| Incomplete outcome data  (attrition bias) | Low risk | Comment: The consort flow chart of the study showed 2 patients in the IV iron group and 3 patients in the placebo group were lost to follow-up, which was between the two treatment groups |
| Selective reporting  (reporting bias) | Low risk | Comment: Important clinical outcomes including blood transfusion rate, and hemoglobin level changes were reported. |
| Other potential source of bias | Low risk | Comment: no authors’ financial conflict of interest was identified. |

**Table 5: Johansson et al. 2015**

| ***Methods*** | ***Participants characteristics*** | |
| --- | --- | --- |
| Country and year | Denmark 2015 | |
| Type of surgery | Cardiac surgery | |
| Participants number: Intravenous iron/Placebo | 30/30 | |
| Post-randomization dropout(s): | In the patient disposition chart, they mentioned that four patients from the IV group discontinued the study (three were non-compliant and one patient retracted his consent). Five patients from the placebo group discontinued the trial due to non-compliance. However, the participant discontinuation was comparable between the two treatment groups (group A: 13.3% (4/ 30); group B: 16.7% (5/30)). | |
| Main age (y): Intravenous iron/ Placebo | 65 ± 8/ 65 ± 11 | |
| Female sex (%)  Intravenous iron: Placebo | 13: 13 | |
| Control arm: | Placebo | |
| Inclusion criteria: | Adult patients undergoing elective or subacute coronary artery bypass grafting (CABG), valve replacement or a combination with a Hb ≥12.0 g/dl for women and a Hb ≥13.0 g/dl for men, and who were willing to provide written informed consent. | |
| Exclusion criteria: | Patients with iron overload or disturbances in utilization of iron (e.g. hemochromatosis and hemosiderosis), s-ferritin >800 ng/ml, known hypersensitivity to any excipients in the investigational drug products, history of multiple allergies, decompensated liver cirrhosis and hepatitis, alanine aminotransferase >3 times normal upper value, acute infections, rheumatoid arthritis with symptoms or signs of active joint inflammation, pregnant or nursing women, participation in any other clinical trial where the trial drug had not passed five half-lives prior to screening, untreated vitamin B12 or folate deficiency, other IV or oral iron treatment within four weeks prior to screening visit, erythropoietin treatment within four weeks prior to screening visit, and impaired renal function defined by serum creatinine >150 μmol/L. Patients who received blood transfusion <30 days before screening and/or during the surgery were excluded from participation in the trial. | |
| Intervention | Participants were randomly allocated to one of the following groups:  Group A: IV iron arm who received isomaltoside 1000 mg as a single-dose infusion of 1000 mg over 15 min with a maximum single dose of 20 mg/kg.  Group B: placebo arm who received saline (Natriumklorid 9 mg/ml; Fresenius Kabi, Copenhagen, Denmark) as a single-dose infusion of 100 ml over 15 min. | |
| Trial’s outcomes reported and related to our primary outcomes | Outcomes reported were change in hemoglobin levels and blood transfusion requirements. | |
| Time of outcomes measurements | Hemoglobin and ferritin levels were measured at one day before surgery or same day, at day five post-surgery and four weeks postoperatively. Transfusion requirement was measured post-surgery till four weeks postoperatively. | |
| Last follow up time | Four weeks postoperatively. | |
| Additional notes | The trial recruited only non-anemic patients.  Attempts to contact the trial authors to request more data were unsuccessful. | |
| ***Risk of bias assessment*** | | |
| Risk of bias item | Authors’ judgement | Authors’ backing for judgement |
| Random sequence generation  (selection bias) | Low risk | Quote: “The randomization list was prepared centrally by a contract research organization, Max Neeman International Data Management Centre, using a validated computer program (Statistical Analysis Software [SAS] 9.1.3; SAS Institute Inc., Cary, NC) PROC PLAN procedure. An interactive web response system method was used to randomize the eligible patient to the treatment groups” |
| Allocation concealment  (selection bias) | Low risk | Quote: “When the patient data had been entered into the interactive web response system, a unique randomization number was generated for the patient, identifying which treatment the patient was allocated to. The screening and enrolment of the patients were performed by the investigator at the site, whereas the entering of the patient data into the interactive web response system generating the randomization number was typically performed by the trial nurse or trial coordinator.” |
| Blinding of participants and researchers (performance bias) | Low risk | Quote: “The screening and enrolment of the patients were performed by the investigator at the site, whereas the entering of the patient data into the interactive web response system generating the randomization number was typically performed by the trial nurse or trial coordinator” |
| Blinding of outcome assessment  (detection bias) | Low risk | Comment: not stated, however, hemoglobin measurement and transfusion rate as objective outcome measurement are unlikely to be influenced. |
| Incomplete outcome data  (attrition bias) | Low risk | Comment: In the patient disposition chart, they mentioned that 1 patient withdraw his consent, 4 patients from the IV group and 5 patients from the placebo group discontinued the trial. The discontinuation was comparable between the two trial groups (group A: 13 3% (4/ 30); group B: 16 7% (5/30)) |
| Selective reporting  (reporting bias) | Low risk | Comment: Important clinical outcomes including blood transfusion rate, and hemoglobin level changes were reported. |
| Other potential source of bias | High risk | Quote: (Department of Clinical Immunology, Copenhagen University Hospital, Rigshospitalet) received a fee per patient.” and  Quote: “The study was funded by Pharmacosmos A/S (Holbaek, Denmark)” |

**Table 6: Keeler et al. 2017**

| *Methods* | *Participants characteristics* | |
| --- | --- | --- |
| Country | UK | |
| Type of surgery | Colorectal cancer resection | |
| Participants number: Intravenous iron/oral iron | 55/61 | |
| Post-randomization dropout(s): | Two patients in intravenous iron group and four patients in oral iron group did not have surgical resection | |
| Main age (y): Intravenous iron/oral iron | Median (IQR) 73⋅8 (67⋅4–78⋅6)/74⋅7 (67⋅9–80⋅8) | |
| Female sex (%)  Intravenous iron: Oral iron | 39: 36 | |
| Control arm: | Oral iron | |
| Inclusion criteria: | Anemic patients (at least 10 g/L of hemoglobin below the WHO definition of anemia) who are medically fit for surgery, willing to participate and undergoing elective bowel resection surgery for colorectal cancer without metastasis. Their surgery has to be scheduled at least 14 days from date of planned initiation of iron infusion. Participant has to be willing to allow general practitioner and consultant to be notified of participation in the study. | |
| Exclusion criteria: | Female patients who were pregnant, lactating or planning a pregnancy during the course of the study. Patients with known blood, kidney, liver disease, iron overload, or allergic to intravenous iron or related iron products. Anemic patients necessitating urgent transfusion or had planned blood donation during the study, or who had prior gastric or intestinal operation (where ≥ 50% of stomach or terminal ileum has been resected). Patients requiring urgent operation or unable to consent. Other significant disease or medical disorder which, in the opinion of the investigator, may either put the participants at risk because of participation in the study, or may influence the result of the study, or the participants’ ability to participate in the trial. Participants who have participated in another research study involving an investigational medical product in the past 12 weeks or prisoners. | |
| Intervention: | Participants were randomly allocated to one of the following groups:  Group 1: IV iron arm who received ferric carboxymaltose with a maximum dose of 1000 mg per week and a maximum of 2000 mg during the trial. The total dose was ranged between 1000-2000 mg according to the body weight and the hemoglobin level. If patients required two doses, the second dose was administered at least 7 days after the first dose.  Group 2: oral iron arm who received ferrous sulphate 200 mg twice daily and was continued until surgery. | |
| Trial’s outcomes reported and related to our primary outcomes | Outcomes reported were change in hemoglobin levels and blood transfusion requirements. | |
| Time of outcomes measurements: | Hemoglobin levels were measured at recruitment, pre-surgery surgery (on the day of surgery), and post-surgery. Transfusion requirement was measured from the recruitment time till post-surgery. | |
| Last follow-up time: | As a routine postoperative outpatient follow-up appointment about 2–3 months post-hospital discharge without reporting hemoglobin or transfusion data at this visit. | |
| Additional notes: | The trial recruited anemic patients.  Attempts to contact the trial authors to request more data were unsuccessful. | |
| ***Risk of bias assessment*** |  | |
| Risk of bias item | Authors’ judgement | Authors’ backing for judgement |
| Random sequence generation  (selection bias) | Low risk | Quote: "Recruited patients were randomized in a 1:1 fashion via a web-based system using variable block allocation, stratified by patient sex and age." |
| Allocation concealment  (selection bias) | Low risk | Quote: "Recruited patients were randomized in a 1:1 fashion via a web-based system using variable block allocation, stratified by patient age and sex"  Comment: it was computer-generated random allocation |
| Blinding of participants and researchers (performance bias) | Low risk | Quote: “Treatment allocation was open-label as it was not possible to conceal the darkening of stool when ingesting oral iron.”  Comment: blinding of the participants was not feasible because they were receiving either injection or pill. Also, darkening of stool happens due to ingestion of oral iron. However, this would be unlikely to affect measurement on objective quantitative outcomes as transfusion, hemoglobin level, serum ferritin or transferrin saturations %. |
| Blinding of outcome assessment  (detection bias) | Low risk | Comment: not stated, however, hemoglobin measurement and transfusion rate as objective outcome measurement are unlikely to be influenced. |
| Incomplete outcome data  (attrition bias) | Low risk | Comment: while four patients had their operations cancelled on the surgery day due to clinical condition, one patient died during anesthesia induction, and one was deemed inoperable at laparotomy. However, the trial analysis plan was an intention-to-treat one. |
| Selective reporting  (reporting bias) | Low risk | Comment: Important clinical outcomes including blood transfusion rate, and hemoglobin level changes were reported. |
| Other potential source of bias | Low risk | Comment: no authors’ financial conflict of interest was identified. |

**Table 7: Kim et al. 2009**

| ***Methods*** | ***Participants characteristics*** | |
| --- | --- | --- |
| Country and year | South Korea 2009 | |
| Type of surgery | Gynecological surgery for menorrhagia | |
| Participants number: Intravenous iron/oral iron | 30/26 | |
| Post-randomization dropout(s): | Nine (23.1%) patients in the intravenous group and 11(29.7%) patients in the oral iron group were not included in the trial analysis due to treatment compliance of 80% or less. | |
| Main age (y): Intravenous iron/oral iron | 42 .0 ± 7.4/42.3 ± 8.0 | |
| Female sex (%)  Intravenous iron: oral iron | 100 | |
| Control arm: | Oral iron | |
| Inclusion criteria: | Patients having menorrhagia with hemoglobin levels below 9.0 g/dL, diagnosed as iron deficiency anemia and scheduled to undergo surgical treatment. | |
| Exclusion criteria: | Anemia from causes other than iron deficiency.  Current administration of iron.  Previous iron treatment or transfusion within three months.  History of hematological disease.  Chronic disease not suitable for clinical trial. | |
| Intervention | Participants were randomly allocated to one of the following groups:  Group 1: IV iron arm who received 200 mg iron sucrose every other day, three times a week starting three weeks prior to surgery using a dose based on the Ganzoni formula to calculate the total body iron deficit until calculated dose was achieved.  Group 2: oral iron arm who received two ampoules of protein succinylate (80 mg of elementary iron per day) beginning three weeks before surgery. | |
| Trial’s outcomes reported and related to our primary outcomes | Outcomes reported were change in hemoglobin levels. Authors did not report transfusion data. | |
| Time of outcomes measurements | Hemoglobin levels were measured pre-treatment and just prior to surgery. | |
| Last follow up time | Hospital discharge | |
| Additional notes | The trial recruited anemic patients only.  Attempts to contact the trial authors to request more data were unsuccessful. | |
| ***Risk of bias assessment*** | | |
| Risk of bias item | Authors’ judgement | Authors’ backing for judgement |
| Random sequence generation  (selection bias) | Low risk | Quote: “According to the computer-generated randomization table, the participants were randomly assigned to either the intravenous iron sucrose (Venoferrum; Vifor International, Ltd, St. Gallen Switzerland) or the oral iron protein succinylate (Hemo-Q Soln; Italfarmaco SpA, Milan. Italia) treatment group” |
| Allocation concealment  (selection bias) | Low risk | Quote: “Group allocation was determined by one of the authors who was not involved in patient care. As each patient gave consent for the study, the patient was consecutively assigned to 1 of the 2 treatment groups according to the number of a randomization table” |
| Blinding of participants and researchers (performance bias) | Low risk | Comment: blinding of the participants was not feasible because they were receiving either injection or pill. However, the trial outcome as hemoglobin level change was an objective one, which was unlikely to be impacted. |
| Blinding of outcome assessment  (detection bias) | Low risk | Comment: no blinding, however, hemoglobin measurement is unlikely to be Influenced. |
| Incomplete outcome data  (attrition bias) | High risk | Quote: "Participants who had > 80% compliance were included in the analysis"  Comment: Post-randomization dropout(s): 20 (26.3%) due to compliance less than 80%.  Comment: as the trial analysis was intention-to-treat method, this was crucial since oral iron therapy is frequently associated with poor tolerance, and consequently more poor compliance exist. |
| Selective reporting  (reporting bias) | High risk | Comment: Important clinical outcomes as blood transfusion rates were not reported. |
| Other potential source of bias | Low risk | Comment: no authors’ financial conflict of interest was identified. |

**Table 8: Serrano-Trenas et al. 2011**

| ***Methods*** | ***Participants characteristics*** | |
| --- | --- | --- |
| Country and year | Spain 2011 | |
| Type of surgery | Hip fracture surgery in elderly patients | |
| Participants number: Intravenous iron/control | 99/97 | |
| Post-randomization dropout(s): | In the patient flow diagram, authors mentioned that in the intravenous iron group, 11 patients died (one patient before surgery, five patients before discharge, and five patients before check-up). In the control group, 10 patients died (three patients before surgery, five patients before discharge, and two patients before check-up). | |
| Main age (y): Intravenous iron/control | 83.46 ± 7.1/82.53 ± 6 .4 | |
| Female sex (%)  Intravenous iron: control | 80/79 | |
| Control arm: | The standard protocolized treatment | |
| Inclusion criteria: | Elderly patients (aged over 65) undergoing hip fracture surgery. | |
| Exclusion criteria: | Patients diagnosed before the admission with iron overload disorders, hyper- sensitivity to oral or parenteral iron preparations, asthma or other severe atopic, active infection or neoplasm. Patients receiving clopidogrel or a dose >150 mg/24 hour of acetylsalicylic acid. Patient with no surgical indication for the current fracture, disorders impaired coagulation (partial thromboplastin time >2.5%, international normalized ratio (INR) >1.5), liver disorders with elevated trans- aminases (aspartase aminotransferase [AST] >70 U/L, alanine aminotransferase [ALT] >55 U/L), and chronic kidney failure (creatinine >2 mg/dL) or patients on dialysis. | |
| Intervention | Participants were randomly allocated to one of the following groups:  Group A: Control arm who received the standard protocolized treatment.  Group B: IV iron arm who received three doses of 200 mg at 48-hour intervals, starting on the day of admission; administration was by slow perfusion of two 100-mg ampoules diluted in 250 mL of 9% saline solution over a 90-minute period. The first dose was administered in the first 24 hours after admission, always before surgical intervention. The following doses were administered before or after surgery, depending on the time of surgery. | |
| Trial’s outcomes reported and related to our primary outcomes | Outcomes reported were change in hematocrit, and blood transfusion requirements. We received the data for the hemoglobin change after contacting the first author. | |
| Time of outcomes measurements | Hematocrit values were measured on admission, at one-day post-surgery, and 7-days postoperatively. Transfusion requirement was measured preoperatively. | |
| Last follow up time | Hospital discharge | |
| Additional notes | The trial recruited anemic and non-anemic patients  We contacted the first author, and he provided us with all hemoglobin data. | |
| ***Risk of bias assessment*** | | |
| Risk of bias item | Authors’ judgement | Authors’ backing for judgement |
| Random sequence generation  (selection bias) | Low risk | Quote: “Randomization lists were generated in blocks of 10 to ensure equal group sizes, and allocation was made using sequentially numbered opaque sealed envelopes”  Comment: Block randomisation in blocks of 10. |
| Allocation concealment  (selection bias) | Low risk | Quote: “………allocation was made using sequentially numbered opaque sealed envelopes, so that neither the patient nor the investigator could know which group the subject was assigned to before his or her consent to participation” |
| Blinding of participants and researchers (performance bias) | Low risk | Quote: “Blinding procedures were not used in the trial because they were considered too complex for daily clinical practice; this was compensated for by the rigorous nature of most of the study variables and by the blinded evaluation of trial data by an independent evaluator”  Comment: the outcome measures are objective and unlikely to be influenced by blinding. |
| Blinding of outcome assessment  (detection bias) | Low risk | Quote: "………blinded evaluation of trial data by an independent evaluator" |
| Incomplete outcome data  (attrition bias) | Low risk | Comment: the causes for patient withdrawal reported in each arm, which seem to be balanced, in addition, the data were analyzed using intention-to-treat method. |
| Selective reporting  (reporting bias) | Low risk | Comment: Important clinical outcomes including blood transfusion rate, and hemoglobin level changes were reported. |
| Other potential source of bias | Low risk | Comment: no authors’ financial conflict of interest was identified. |

**Table 9: Shah et al. 2016**

| ***Methods*** | ***Participants characteristics*** | |
| --- | --- | --- |
| Country and year | India 2016 | |
| Type of surgery | Gynecological surgery for menorrhagia | |
| Participants number: Intravenous iron/oral iron | 55/55 | |
| Post-randomization dropout(s): | No dropout happened during the study period and all women were followed regularly and assessed for side effects, compliance, clinical and laboratory response. | |
| Main age (y): Intravenous iron/oral iron | Majority of women were in the age group of 40-49 years (59% and 57% in group A (intravenous iron) and B (oral iron) respectively). | |
| Female sex (%)  Intravenous iron: Oral iron | 100 | |
| Control arm: | Oral iron. | |
| Inclusion criteria: | Patients with hemoglobin less than 10 gm/dl; features of iron deficiency were evidenced by hypochromic microcytic anemia, low MCV, MCH and MCHC values, increased RDW, low serum iron and serum ferritin levels; and women having their surgical procedure at least three to four weeks after preoperative assessment. | |
| Exclusion criteria: | Exclusion criteria were anemia other than iron deficiency; those women requiring early surgery, i.e., women with malignancy; and those unwilling to participate in the study. | |
| Intervention | Participants were randomly allocated to one of the following groups:  Group A: IV iron arm who received 100 mg (2 ampoules) in 100 ml normal saline by slow IV infusion. The dose was repeated on alternate day basis until target hemoglobin of 10 g/dL was achieved. The treatment either with IV iron or oral iron starts 4-weeks pre-surgery.  Group B: Control arm who received ferrous sulphate as oral iron in the dose of one tablet (200 mg salt) three times a day. | |
| Trial’s outcomes reported and related to our primary outcomes | Outcomes reported were change in hemoglobin levels. Authors did not report transfusion data. | |
| Time of outcomes measurements | Hemoglobin values were measured pre-treatment and post-treatment (pre-surgery). | |
| Last follow up time | Hospital discharge | |
| Additional notes | The trial recruited anemic patients.  Attempts to contact the trial authors to request more data were unsuccessful. | |
| ***Risk of bias assessment*** | | |
| Risk of bias item | Authors’ judgement | Authors’ backing for judgement |
| Random sequence generation  (selection bias) | Low risk | Quote: “Women matching the above criteria, allocated to group A (IV iron sucrose) or group B (oral iron) by sequentially numbered opaque envelope pre- pared by the person not involved in the study” |
| Allocation concealment  (selection bias) | Low risk | Quote: “Women matching the above criteria, allocated to group A (IV iron sucrose) or group B (oral iron) by sequentially numbered opaque envelope pre- pared by the person not involved in the study” |
| Blinding of participants and researchers (performance bias) | Low risk | Comment: blinding of the participants was not feasible because they were receiving either injection or pill. However, the trial outcome as hemoglobin level change was an objective one, which was unlikely to be impacted. |
| Blinding of outcome assessment  (detection bias) | Low risk | Comment: no blinding, however, hemoglobin measurement is unlikely to be Influenced. |
| Incomplete outcome data  (attrition bias) | Low risk | Quote: “No dropout happened during the study period and all women were followed regularly and assessed for side effects, compliance, clinical and laboratory response.” |
| Selective reporting  (reporting bias) | High risk | Comment: Important clinical outcomes as blood transfusion rates were not reported. |
| Other potential source of bias | Low risk | Quote: “Financial Support: None declared” and “Conflict of interest: None declared” |

**Table 10: Weisbach et al. 1999**

| ***Methods*** | ***Participants characteristics*** | |
| --- | --- | --- |
| Country and year | Germany 1999 | |
| Type of surgery | Major orthopedic or cardiovascular surgery | |
| Participants number: Intravenous iron/control | 30/30 | |
| Post-randomization dropout(s): | Quote: “Of these 123 patients, 90 completed the study. Eight patients dropped out of the study because of acute viral and bacterial infections, two patients did not take oral iron as planned, eight patients refused to continue for reasons unrelated to iron medication, and four patients developed unstable angina or cardiac insufficiency. In 7 patients, ABD could not be continued as planned because of insufficient vein access or severe vasovagal reactions. Four patients dropped out of the study because of suspected side effects of iron therapy as described below. Overall, 13 patients were excluded from Group 2, and nine from Group 3. Finally, each of the three treatment groups consisted of 15 females and 15 male patients. One female patient in Group 3 who completed ABD did not undergo surgery. This patient was included in the analysis of the preoperative period”. | |
| Main age (y): Intravenous iron/control | 64.4 ± 14.7/64.1 ± 9.5 | |
| Female sex (%)  Intravenous iron: control | 50: 50 | |
| Control arm: | The standard protocolized treatment | |
| Inclusion criteria: | Adult patients (>18years old) scheduled for autologous blood transfusion from Day -49 to Day -35 before major orthopedic or cardiovascular surgery, with a minimum of 3 autologous units scheduled for collection. | |
| Exclusion criteria: | Patients with general contraindications of autologous blood donation (ABD), evidence of blood loss, renal disease, chronic and acute inflammatory or malignant disorders, pregnancy, lactation or inadequate contraception, myelotoxic therapy as evaluated by clinical history, body weight under 50 kg, initial ferritin <20 μg per L, and a C-reactive protein level over 0.9 mg per dL, or occult gastrointestinal blood loss. | |
| Intervention | Participants were randomly allocated to one of the following groups  Group 1: IV iron arm who received 200 mg of iron sucrose, given after each donation. At enrollment, at least one week before the first donation, an initial IV iron dose was given to fill iron stores, to optimize iron availability in patients with Hb level below 150 g/L.  Group 2: control arm that received no iron medication. | |
| Trial’s outcomes reported and related to our primary outcomes | Outcomes reported were change in hemoglobin levels and blood transfusion requirements. | |
| Time of outcomes measurements | Hemoglobin was measured at enrollment, at every donation, at surgery, and at hospital discharge. Transfusion requirement was measured postoperatively. | |
| Last follow up time | Hospital discharge | |
| Additional notes | The trial recruited non-anemic patients.  Attempts to contact the trial authors to request more data were unsuccessful. | |
| ***Risk of bias assessment*** | | |
| Risk of bias item | Authors’ judgement | Authors’ backing for judgement |
| Random sequence generation  (selection bias) | Low risk | Quote: “The trial medication was assigned to patients on the basis of their chronologic enrollment and the sequential order of the trial medication in a randomization list, determined by drawing lots before the start of the study” |
| Allocation concealment  (selection bias) | Low risk | Quote: “The trial medication was assigned to patients on the basis of their chronologic enrollment and the sequential order of the trial medication in a randomization list, determined by drawing lots before the start of the study” |
| Blinding of participants and researchers (performance bias) | Low risk | Comment: the outcome measures are objective and unlikely to be influenced by blinding. |
| Blinding of outcome assessment  (detection bias) | Low risk | Comment: not stated, however, this would be unlikely to affect measurement on objective quantitative outcomes as transfusion rate and hemoglobin level. |
| Incomplete outcome data  (attrition bias) | Low risk | Quote: “Of these 123 patients, 90 completed the study. Eight patients dropped out of the study because of acute viral and bacterial infections, 2 patients did not take oral iron as planned, 8 patients refused to continue for reasons unrelated to iron medication, and 4 patients developed unstable angina or cardiac insufficiency. In 7 patients, ABD could not be continued as planned because of insufficient vein access or severe vasovagal reactions. Four patients dropped out of the study because of suspected side effects of iron therapy as de- scribed below. Overall, 13 patients were excluded from Group 2, 11 from Group 1, and 9 from Group 3. Finally, each of the three treatment groups consisted of 15 female and 15 male patients. One female patient in Group 3 who completed ABD did not undergo surgery. This patient was included in the analysis of the preoperative period” |
| Selective reporting  (reporting bias) | Low risk | Comment: Important clinical outcomes including blood transfusion rate, and hemoglobin level changes were reported. |
| Other potential source of bias | High risk | Quote: “Supported in part by Vifor (International), Inc”  Comment: this might introduce funding bias. Also, the authors did not declare their conflict of interest. |
