# Supplementary material for: Role of preoperative intravenous iron therapy to correct anemia before major surgery: a systematic review and meta-analysis
Source: Syst Rev. 2021 Jan 23;10:36. doi: 10.1186/s13643-021-01579-8 (PMC7824930; doi:10.1186/s13643-021-01579-8)
Supplement: Supplementary file 2 — Additional file 2. A: Forest-plots for the Sensitivity Analysis. B: Forest-plots for the Subgroup Analysis. C: Forest-plots for Hemoglobin Values at Different Time Points. D: Forest-plots for the Different Iron-deficiency Anemia Blood Tests. E: Forest-plots for the Safety Endpoints of Intravenous Iron versus Placebo or Oral Iron. [file 13643_2021_1579_MOESM2_ESM.docx]

ADDITIONAL FILE 2

**Additional File 2-A: Forest-plots for the Sensitivity Analysis**

**Figure 1: Forest plot comparison shows the pooled comparison effect of intravenous iron therapy versus placebo/standard of care groups on the proportion of the transfused patients after exclusion of Weisbach trial as a sensitivity analysis (random effects model).**

**Figure 2: Forest plot comparison shows the effect of intravenous iron therapy versus placebo/standard of care groups on the occurrence of associated non-serious adverse effects after including a trial (Garrido-Martín et al.) with zero events (random effects model).**

**Figure 3: Forest plot comparison shows the pooled comparison effect of intravenous iron therapy versus placebo/standard of care groups on the 30-day mortality after including a trial (Johansson et al.) with zero events (random effects model).**

**Additional File 2-B: Forest-plots for the Subgroup Analysis**

**Figure 1: Forest plot comparison shows the pooled comparison effect of intravenous iron therapy versus placebo/standard of care groups on the proportion of the transfused patients with the different surgeries as a subgroup analysis (random effects model).**

**Figure 2: Forest plot comparison shows the pooled comparison effect of intravenous iron therapy versus placebo/standard of care groups on the proportion of the transfused patients as a subgroup analysis for different intravenous iron preparations (random effects model).**

**Figure 3: Forest plot comparison shows the pooled comparison effect of intravenous iron therapy versus placebo/standard of care groups on the proportion of the transfused patients as a subgroup analysis for intravenous iron injection timing (random effects model).**

**Additional File 2-C: Forest-plots for Hemoglobin Values at Different Time Points**

**Figure 1: Forest plot comparison shows the pooled comparison of baseline value of hemoglobin level (g/L) between intravenous iron therapy versus placebo/standard of care groups (random effects model).**

**Figure 2: Forest plot comparison shows the pooled comparison effect of intravenous iron therapy versus placebo/standard of care groups on the change of hemoglobin level (g/L) at postoperative day # 1 (random effects model).**

**Figure 3: Forest plot comparison shows the pooled comparison effect of intravenous iron therapy versus placebo/standard of care groups on the change of hemoglobin level (g/L) at the hospital discharge (random effects model).**

**Additional File 2-D: Forest-plots for the Different Iron-deficiency Anemia Blood Tests**

**Figure 1: Forest plot comparison shows the pooled comparison of baseline value of ferritin levels (ng/mL) between intravenous iron therapy versus placebo/standard of care groups (random effects model).**

**Figure 2: Forest plot comparison shows the pooled comparison effect of intravenous iron therapy versus placebo/standard of care groups on the ferritin levels (ng/mL) at the post-treatment (pre-surgery) time (random effects model).**

**Figure 3: Forest plot comparison shows the pooled comparison effect of intravenous iron versus oral iron groups on the ferritin levels (ng/mL) at the post-treatment (pre-surgery) time (random effects model).**

**Figure 4: Forest plot comparison shows the pooled comparison effect of intravenous iron therapy versus placebo/standard of care groups on the ferritin levels (ng/mL) at the hospital discharge time (random effect model).**

**Figure 5: Forest plot comparison shows the pooled comparison effect of intravenous iron therapy versus placebo/standard of care groups on the ferritin levels (ng/mL) as follow-up > 4 weeks postoperatively (random effect model).**

**Figure 6: Forest plot comparison shows the pooled comparison of baseline value of the reticulocyte percentage (%) between intravenous iron therapy versus placebo/standard of care groups (random effects model).**

**Figure 7: Forest plot comparison shows the effect of intravenous iron therapy versus placebo/standard of care groups on the reticulocyte percentage (%) at the post-treatment (pre-surgery) time.**

**Figure 8: Forest plot comparison shows the effect of intravenous iron therapy versus placebo/standard of care groups on the reticulocyte percentage (%) at the hospital discharge time.**

**Figure 9: Forest plot comparison shows the effect of intravenous iron therapy versus placebo/standard of care groups on the reticulocyte (%) percentage at a month from hospital discharge.**

**Figure 10: Forest plot comparison shows the pooled comparison of baseline value of the mean corpuscular volume (MCV) level (fL) between intravenous iron versus placebo/standard of care groups (random effects model).**

**Figure 11: Forest plot comparison shows the pooled comparison effect of intravenous iron versus placebo/standard of care groups on the mean corpuscular volume (MCV) level (fL) at post-treatment (pre-surgery) time (random effects model).**

**Figure 12: Forest plot comparison shows the pooled comparison of baseline value of the hematocrit percentage (%) between intravenous iron therapy versus placebo/standard of care groups (random effects model).**

**Figure 13: Forest plot comparison shows the pooled comparison effect of intravenous iron therapy versus placebo/standard of care groups on the hematocrit value (%) at postoperative day # 1 (random effects model).**

**Figure 14: Forest plot comparison shows the pooled comparison effect of intravenous iron therapy versus placebo/standard of care groups on the hematocrit value (%) at the hospital discharge (random effect model).**

**Figure 15: Forest plot comparison shows the pooled comparison of baseline value of the transferrin saturation value (TSAT %) between intravenous iron therapy versus placebo/standard of care groups (random effect model).**

**Figure 16: Forest plot comparison shows the pooled comparison effect of intravenous iron therapy versus placebo/standard of care groups on the transferrin saturation value (TSAT %) at the post-treatment (pre-surgery) time (random effects model).**

**Figure 17: Forest plot comparison shows the pooled comparison effect of intravenous iron therapy versus placebo/standard of care groups on the transferrin saturation value (TSAT %) at the hospital discharge time (random effects model).**

**Figure 18: Forest plot comparison shows the pooled comparison of baseline value of the mean corpuscular hemoglobin (MCH) level (pg/cell) between intravenous iron therapy versus placebo/standard of care groups (random effects model).**

**Figure 19: Forest plot comparison shows the pooled comparison effect of intravenous iron therapy versus placebo/standard of care groups on the mean corpuscular Hb (MCH) level (pg/cell) at the post-treatment (pre-surgery) time (random effects model).**

**Figure 20: Forest plot comparison shows the comparison of baseline value of the mean corpuscular hemoglobin concentration (MCHC) level (g/L) between intravenous iron therapy versus placebo/standard of care groups**.

**Figure 21: Forest plot comparison shows the effect of intravenous iron therapy versus placebo/standard of care groups on the mean corpuscular hemoglobin concentration (MCHC) level (g/L) at the post-treatment (pre-surgery) time.**

**Figure 22: Forest plot comparison shows the pooled comparison of baseline value of serum iron level (μmol/l) between intravenous iron therapy versus placebo/standard of care groups (random effects model).**

**Figure 23: Forest plot comparison shows the effect of intravenous iron therapy versus placebo/standard of care groups on the change of serum iron level (μmol/l) at the post-treatment (pre-surgery) time.**

**Figure 24: Forest plot comparison shows the effect of intravenous iron therapy versus placebo/standard of care groups on the change of serum iron (μmol/l) at the hospital discharge time.**

**Additional File 2-E: Forest-plots for the Safety Endpoints**

**of Intravenous Iron versus Placebo or Oral Iron**

**Figure 1: Forest plot comparison shows the effect of intravenous iron therapy versus placebo/standard of care groups on the occurrence of associated non-serious adverse effects (random effects model).**

**Figure 2: Forest plot comparison shows the effect of intravenous iron therapy versus placebo/standard of care on groups on the occurrence of associated serious adverse effects.**

**Figure 3: Forest plot comparison shows the pooled comparison effect of intravenous iron therapy versus placebo/standard of care groups on the 30-day mortality (random effects model).**

**Figure 4: Forest plot comparison shows the effect of intravenous iron therapy versus placebo/standard of care groups on the mortality ≥ 2-months post-hospital discharge.**

**Figure 5: Forest plot comparison shows the pooled comparison effect of intravenous iron versus placebo/standard of care groups on the postoperative infection occurrence (random effects model).**
