# Supplementary material for: Role of preoperative intravenous iron therapy to correct anemia before major surgery: a systematic review and meta-analysis
Source: Syst Rev. 2021 Jan 23;10:36. doi: 10.1186/s13643-021-01579-8 (PMC7824930; doi:10.1186/s13643-021-01579-8)
Supplement: Supplementary file 3 — Additional file 3. Summary of Findings Table. [file 13643_2021_1579_MOESM3_ESM.docx]

| Additional File 11: Summary of findings table for the main outcomes | | | | | |  |  |  |
| --- | --- | --- | --- | --- | --- | --- | --- | --- |
| Intravenous iron compared to placebo/standard of care for preoperative surgical patients | | | | | |  |  |  |
| **Patient or population: preoperative surgical patients**  **Setting: Major surgery**  **Intervention: Intravenous iron**  **Comparison: placebo/standard of care** | | | | | |  |  |  |
| **Outcome** | Anticipated absolute effects (95% CI) | | Relative effect (95% CI) | № of participants  (studies) | Certainty | What happens | |  |
|  | Risk with placebo/standard of care | Risk with Intravenous iron |  |  |  |  |  |  |
| **Total proportion of the transfused patients in each group** | 402 per 1,000 | **338 per 1,000** (285 to 398) | **RR 0.84** (0.71 to 0.99) | 873 (8 RCTs) | ⨁⨁⨁◯ MODERATE ^a,b^ | A sensitivity analysis with exclusion of one trial (Weisbach et al 1999), which was designed mainly for autotransfusion before going to major surgery showed higher risk reduction (17%) in favor of the intravenous iron group. This increase was more statistically insignificant. | |  |
| **Haemoglobin level post-treatment and pre-surgery (g/dL).** | The mean outcome Hemoglobin level post-treatment/pre-surgery (g/d). was **0** | MD **7.15 higher** (2.26 higher to 12.04 higher) | - | 580 (7 RCTs) | ⨁⨁⨁◯ MODERATE ^a,c,d, e^ | A sensitivity analysis with exclusion of one trial (Edwards et al 2009) for which we had to estimate the standard deviation, the mean difference increased to be7.93 g/dl and became more statistically insignificant. | |  |
| **Haemoglobin level at post-operative day # 1(g/dL)** | The mean hemoglobin level at post-operative day # 1(g/dL) was **0** | MD -**0.54 higher** (-2.73 lower to 1.66 higher) | - | 565 (4 RCTs) | ⨁⨁⨁◯ MODERATE ^e,f^ | A sensitivity analysis with exclusion of one trial (Edwards et al 2009) for which we had to estimate the standard deviation; the mean difference increased to be -0.52 g/dl. However, this difference was statistically insignificant. | |  |
| **Hemoglobin level at hospital discharge time** | The mean hemoglobin level at hospital discharge time was **0** | MD **0.66 lower** (-1.60 lower to 2.92 higher) | - | 697 (5 RCTs) | ⨁⨁⨁◯ ^MODERATE e,g^ | A sensitivity analysis with exclusion of one trial (Edwards et al 2009) for which we had to estimate the standard deviation; the mean difference decreased to be 0.02 g/dl. However, this difference was statistically insignificant. | |  |
| **Hemoglobin level as follow up >4 weeks post-surgery** | The mean hemoglobin level as follow up >4 weeks post-surgery was **0** | MD **6.46 higher** (3.10 higher to 9.81 higher) | - | 441 (4 RCTs) | ⨁⨁⨁◯ MODERATE ^d,e,h^ | A sensitivity analysis with exclusion of two trials (Garrido-Martin et al 2012 and Johansson et al 2015), which had an unclear risk of bias, the mean difference increased to be 7.61 g/dl and became more statistically significant. | |  |
| **Ferritin level at post-treatment and pre-surgery** | The mean ferritin level at post-treatment and pre-surgery was **0** | MD **94.09 higher** (51.57, 136.61higher to 136.61 higher) | - | 176 (3 RCTs) | ⨁⨁⨁◯ MODERATE ^e,i^ | A sensitivity analysis with exclusion of one trial (Weisbach et al 1999), which was designed mainly for autotransfusion before going to major surgery, the mean difference increased to be 117.03 ng/mL, but this increase was statistically insignificant. | |  |
| **Ferritin level at hospital discharge** | The mean ferritin level at hospital discharge was **0** | MD **547.77 higher** (36.61 higher to 1058.94 higher) | - | 369 (3 RCTs) | ⨁◯◯◯ VERY LOW ^e,j,k^ | A sensitivity analysis with exclusion of one trial (Edwards et al 2009) for which we had to estimate the standard deviation, the mean difference increased to 820.16 ng/mL and became highly statistically insignificant. In addition, the wide confidence interval became narrower [764.83, 875.49], and the imprecision was improved. | |  |
| **Serious adverse effects** | 300 per 1,000 | **267 per 1,000** (120 to 597) | **RR 0.89** (0.40 to 1.99) | 60 (1 RCT) | ⨁⨁⨁◯ MODERATE ^e,l,m^ | The analysis includes only on trial, which was unique to report on the serious adverse effects. | |  |
| **Non-serious adverse effects** | 84 per 1,000 | **98 per 1,000** (67 to 144) | **RR 1.17** (0.80 to 1.71) | 803 (7 RCTs) | ⨁⨁◯◯ LOW ^e,n,^ | After including the seventh trial (Garrido-Martín et al 2012) that has zero events, the point of estimate and its confidence interval were identical. | |  |
| **Thirty-day mortality** | 57 per 1,000 | **63 per 1,000** (34 to 115) | **RR 1.1** (0.6 to 2.0) | 647 (5 RCTs) | ⨁⨁⨁⨁ HIGH ^e,o^ | After including the fifth trial (Johansson et al 2015) that has zero events, the point of the estimate of 30-day mortality and its confidence interval were identical. | |  |
| *The risk in the intervention group (and its 95% confidence interval) is based on the assumed risk in the comparison group and the relative effect of the intervention (and its 95% CI).   CI: Confidence interval; RR: Risk ratio; MD: Mean difference | | | | | |  |  |  |
| GRADE Working Group grades of evidence High certainty: We are very confident that the true effect lies close to that of the estimate of the effect Moderate certainty: We are moderately confident in the effect estimate: The true effect is likely to be close to the estimate of the effect, but there is a possibility that it is substantially different Low certainty: Our confidence in the effect estimate is limited: The true effect may be substantially different from the estimate of the effect Very low certainty: We have very little confidence in the effect estimate: The true effect is likely to be substantially different from the estimate of effect | | | | | |  |  |  |

Explanations

a. Four of the eight trials included in the analysis had a high risk of bias in one or two domains.

b. Publication bias could not be explored because fewer than 10 trials reported the outcome.

c. One trial (Kim et al 2009) of these seven studies reported the outcome at this time point had a high risk of bias by the exclusion of those participants with less than 80% compliance with treatment.

d. Although the heterogeneity was significant, the dose and/or the timing of the intravenous iron injection can explain this heterogeneity.

e. Publication bias could not be explored because fewer than 10 trials reported the outcome at this time point.

f. Two of the four trials included in the analysis had a high risk of bias in one or two domains.

g. Three of the six trials in the analysis had a high risk of bias in one or two domains.

h. Two of the four included trials in the analysis had a high risk of bias in only one domain.

i. All the three of the six trials included in the analysis had a high risk of bias in one or two domains.

j. Two of the three trials included in the analysis had a high risk of bias in one or two domains.

k. Edwards et al 2009 has inconsistent result with the other two studies results.

l. The only trial that included in the analysis had a high risk of bias in one domain.

m. The confidence interval that overlapped 1 is wide (including the 0.75 and 1.25 that makes the recommendations would vary if the lower versus upper confidence interval were the true estimate.

n. Four of the seven trials included in the analysis had a high risk of bias in one or two domains.

n. Two of the six RCTs had a high risk of bias in two domains and an unclear risk of bias in one domain; a third study had a high risk of bias in one domain.

o. Only study with zero events out of the five trials included in the analysis had a high risk of bias in one or two domains.
